# Floral development and vascularization help to explain merism evolution in *Paepalanthus* (Eriocaulaceae, Poales)

**DOI:** 10.7717/peerj.2811

**Published:** 2016-12-21

**Authors:** Arthur de Lima Silva, Marcelo Trovó, Alessandra Ike Coan

**Affiliations:** 1 Departamento de Botânica, Universidade Estadual Paulista “Júlio de Mesquita Filho”—UNESP, Rio Claro, São Paulo, Brazil; 2 Departamento de Botânica, Universidade Federal do Rio de Janeiro, Rio de Janeiro, Brazil

**Keywords:** Dimery, Floral anatomy, Monocotyledons, Floral ontogeny, Paepalanthoideae

## Abstract

**Background:**

Flowers in Eriocaulaceae, a monocot family that is highly diversified in Brazil, are generally trimerous, but dimerous flowers occur in *Paepalanthus* and a few other genera. The floral merism in an evolutionary context, however, is unclear. *Paepalanthus* encompasses significant morphological variation leading to a still unresolved infrageneric classification. Ontogenetic comparative studies of infrageneric groups in *Paepalanthus* and in Eriocaulaceae are lacking, albeit necessary to establish evolution of characters such as floral merism and their role as putative synapomorphies.

**Methods:**

We studied the floral development and vascularization of eight species of *Paepalanthus* that belong to distinct clades in which dimery occurs, using light and scanning electron microscopies.

**Results:**

Floral ontogeny in dimerous *Paepalanthus* shows lateral sepals emerging simultaneously and late-developing petals. The outer whorl of stamens is absent in all flowers examined here. The inner whorl of stamens becomes functional in staminate flowers and is reduced to staminodes in the pistillate ones. In pistillate flowers, vascular bundles reach the staminodes. Ovary vascularization shows ventral bundles in a commissural position reaching the synascidiate portion of the carpels. Three gynoecial patterns are described for the studied species: (1) gynoecium with a short style, two nectariferous branches and two long stigmatic branches, in most species; (2) gynoecium with a long style, two nectariferous branches and two short stigmatic branches, in *P. echinoides*; and (3) gynoecium with long style, absent nectariferous branches and two short stigmatic branches, in *P. scleranthus*.

**Discussion:**

Floral development of the studied species corroborates the hypothesis that the sepals of dimerous flowers of *Paepalanthus* correspond to the lateral sepals of trimerous flowers. The position and vascularization of floral parts also show that, during dimery evolution in *Paepalanthus*, a flower sector comprising the adaxial median sepal, a lateral petal, a lateral stamen and the adaxial median carpel was lost. In the staminate flower, the outer whorl of staminodes, previously reported by different authors, is correctly described as the apical portion of the petals and the pistillodes are reinterpreted as carpellodes. The occurrence of fused stigmatic branches and protected nectariferous carpellodes substantiates a close relationship between *P.* sect. *Conodiscus* and *P.* subg. *Thelxinoë*. Free stigmatic branches and exposed carpellodes substantiate a close relationship between *P*. sect. *Diphyomene*, *P*. sect. *Eriocaulopsis* and *P*. ser. *Dimeri*. Furthermore, the loss of nectariferous branches may have occurred later than the fusion of stigmatic branches in the clade that groups *P*. subg. *Thelxinoë* and *P*. sect. *Conodiscus*.

## Introduction

Eriocaulaceae is a well-represented monocot family in the Brazilian flora, with approximately 620 species in Brazil (BFG, 2015; Sano et al., 2015). Flowers in Eriocaulaceae are generally trimerous as in most other monocots (Ruhland, 1903; Stützel, 1998). However, dimerous species occur in genera representing both of the two recognized subfamilies, particularly in *Eriocaulon* L. (Eriocauloideae), *Comanthera* L.B.Sm., *Paepalanthus* Mart., and *Syngonanthus* Ruhland (Paepalanthoideae) (Giulietti et al., 2012). Ontogenetic studies suggest that dimerous flowers in Eriocaulaceae may have evolved from trimerous flowers whose median sepal has been suppressed, as it develops late in comparison to the lateral sepals (Stützel, 1984; Stützel, 1985).

*Paepalanthus* is the third largest genus among Brazilian angiosperms (BFG, 2015), comprising more than half of native Eriocaulaceae species (∼340 species) and featuring a great variety of floral patterns and habits (Mabberley, 1987; Giulietti & Hensold, 1990; Giulietti et al., 2012; BFG, 2015). In all species of *Paepalanthus*, the flowers have either free sepals or sepals fused at the base; fused petals in staminate flowers and free petals in pistillate flowers; gynoecium with alternating nectariferous and stigmatic branches, inserted at the same point on the style; and a number of floral parts that depends on whether the flowers are dimerous or trimerous (Ruhland, 1903; Rosa & Scatena, 2003; Rosa & Scatena, 2007; Giulietti et al., 2005; Trovó et al., 2013).

Although some anatomical and ontogenetic studies have been carried out on *Paepalanthus* (Stützel, 1985; Stützel, 1990; Rosa & Scatena, 2007), floral features are still ambiguous within an evolutionary scenario. As in other genera of the family, viz. *Lachnocaulon* and *Syngonanthus* (Stützel & Gansser, 1995), both staminate and pistillate flowers of *Paepalanthus* have similar early stages of development, sharing late-developing petals (Stützel, 1990). Staminate flowers in the genus are isostemonous and lack an outer whorl of staminodes as usual in other representatives of Paepalanthoideae (Rosa & Scatena, 2007). The pistillodes of staminate flowers and the gynoecial nectariferous branches of pistillate flowers were stated as homologous (Rosa & Scatena, 2003; Rosa & Scatena, 2007), but the development of such structures was not studied thoroughly. This overall background is based on a few representatives of *Paepalanthus* and comparative information is still lacking for the infrageneric categories and clades, as well as for other large genera in the family. Körnicke (1863) and Ruhland (1903) initially proposed more than 20 infrageneric categories in *Paepalanthus*, based on its morphology (Trovó et al., 2013). These categories are currently in use and are delimited based on floral characters such as floral merism, conation of floral parts, the shape of pistillodes in staminate flowers, and the presence of bracts subtending the flowers (Ruhland, 1903; Stützel, 1998). In recent phylogenetic studies (Andrade, 2007; Andrade et al., 2010; Giulietti et al., 2012; Trovó et al., 2013), *Paepalanthus* emerges as polyphyletic, and the relationship between its infrageneric categories is unclear, as many of them are also non-monophyletic (Trovó et al., 2013). Recent studies (Giulietti et al., 2012; Trovó et al., 2013) show that dimerous species of *Paepalanthus* occur in five distinct groups: *P*. subg. *Thelxinoë* Ruhland, *P*. sect. *Conodiscus* Ruhland, *P*. sect. *Diphyomene* Ruhland, *P*. sect. *Eriocaulopsis* Ruhland, and *P*. ser. *Dimeri* Ruhland. According to Trovó et al. (2013), dimery has probably evolved multiple times in *Paepalanthus*. However, the available anatomical and ontogenetic studies in dimerous categories of *Paepalanthus* do not help to explain the evolution of floral merism, as the studies are restricted to the anatomy of vegetative organs and the embryology of a few species (Coan & Scatena, 2004; Scatena et al., 2005; Alves, Scatena & Trovó, 2013) and to the floral anatomy of *P. flaccidus* (*P*. sect. *Eriocaulopsis*) (Rosa & Scatena, 2007). Further studies in floral anatomy and ontogeny are required to elucidate the evolution of dimery in the genus and in the family. Furthermore, these studies may help to establish synapomorphies that may contribute to the taxonomy of *Paepalanthus* in a given evolutionary scenario.

## Materials and Methods

We selected eight dimerous species of *Paepalanthus* belonging to *Paepalanthus* subg. *Thelxinoë* Ruhland, *P*. sect. *Conodiscus* Ruhland, *P*. sect. *Diphyomene* Ruhland, *P*. sect. *Eriocaulopsis* Ruhland and *P*. ser. *Dimeri* Ruhland, representing all of the clades of *Paepalanthus* with dimerous flowers proposed by Trovó et al. (2013). The material examined is listed in Table 1, based on the following field permits: SisBio collecting permit no. 47742-1 (February 2015–March 2016) to AL Silva; SisBio permanent permit no. 34782-1 (from May 2012 to present) and collecting permit no. 42939-1 (May 2014–June 2015) to M Trovó; and SisBio permanent permit no. 14929-3 (December 2011 to present) and collecting permits no. 37568-1 and 37568-2 (January 2013–February 2015) to AI Coan.

**Table 1:** Species of *Paepalanthus* examined and respective collections.

Taxa	Collection
***P.* subg. *Thelxinoë***	
*P. scleranthus* Ruhland	Santana do Riacho, Minas Gerais, Scatena et al. 220 (HRCB)
	Santana do Riacho, Minas Gerais, Scatena et al. 267 (HRCB)
	Diamantina, Minas Gerais, Scatena et al. 273 (HRCB)
***P. sect. Conodiscus***	
*P. echinoides* Trovó	Alto Paraíso de Goiás, Goiás, Trovó & Silva 647 (RB)
***P. sect. Diphyomene***	
*P. chiquitensis* Herzog	Alto Paraíso de Goiás, Goiás, Trovó 384 (SPF)
*P. cordatus* Ruhland	Alto Paraíso de Goiás, Goiás, Silva & Trovó 5 (HRCB)
*P. urbanianus* Ruhland	Alto Paraíso de Goiás, Goiás, Borges 708 (SPF)
	Alto Paraíso de Goiás, Goiás, Silva & Trovó 1 (HRCB)
***P* . sect. *Eriocaulopsis***	
*P. flaccidus* (Bong.) Kunth.	Santana do Riacho, Minas Gerais, Scatena et al. 235 (HRCB)
	Santana do Riacho, Minas Gerais, Scatena et al. 240 (HRCB)
	Alto Paraíso de Goiás, Goiás, Silva & Trovó 10 (HRCB)
***P.* ser. *Dimeri***	
*P. elongatus* (Bong.) Körn.	Alto Paraíso de Goiás, Goiás, Borges 706 (SPF)
	Alto Paraíso de Goiás, Goiás, Silva & Trovó 8 (HRCB)
*P. vaginatus* (Bong.) Körn.	Serra da Canastra, Minas Gerais, Echternacht 2596 (HUFU)

**Notes.**

HRCB Universidade Estadual Paulista, Rio Claro, São Paulo, Brazil HUFU Universidade Federal de Uberlândia, Uberlândia, Minas Gerais, Brazil RB Instituto de Pesquisas Jardim Botânico do Rio de Janeiro, Rio de Janeiro, Rio de Janeiro, Brazil SPF Universidade de São Paulo, São Paulo, Brazil

Inflorescences of different developmental stages were collected in regions of “campos rupestres” (rocky outcrops) in the states of Minas Gerais and Goiás, Brazil. The material was fixed using FAA 50 (Johansen, 1940) and then stored in 70% ethanol with a few drops of glycerin.

For the developmental study, the capitula were cut in half or in quarters, and their trichomes were removed using precision tweezers for the observation of floral primordia and young flowers in the central region of the inflorescences. Mature flowers were removed from the peripheral regions of the capitula and then observed in isolation. Samples were dehydrated in ethanol series, critical-point dried, mounted on metal stubs and then coated with gold for observation. SEM images were obtained using a Hitachi TM3000 microscope. The study was carried out in the Laboratório de Microscopia Eletrônica (Instituto de Biociências de Rio Claro, UNESP).

For the study of vascularization, flowers and capitula were dehydrated in a butyl alcohol series and embedded in historesin (Leica Historesin Embedding Kit). Samples were sectioned using glass or steel knives at 3–8 µm on a Leica RM2245 microtome. Sections were stained with periodic acid-Schiff reagent (PAS) (Jensen, 1962) and Toluidine Blue 0.05% 0.1 M in sodium phosphate buffer, pH 6.8 (Feder & O’Brien, 1968), and mounted on permanent slides with Entellan. Photomicrographs were taken using a Leica DM4000 photomicroscope coupled with a DFC450 camera. The study was carried out in the Laboratório de Morfologia Vegetal, Departamento de Botânica (Instituto de Biociências de Rio Claro, UNESP).

## Results

**Figure 1:**
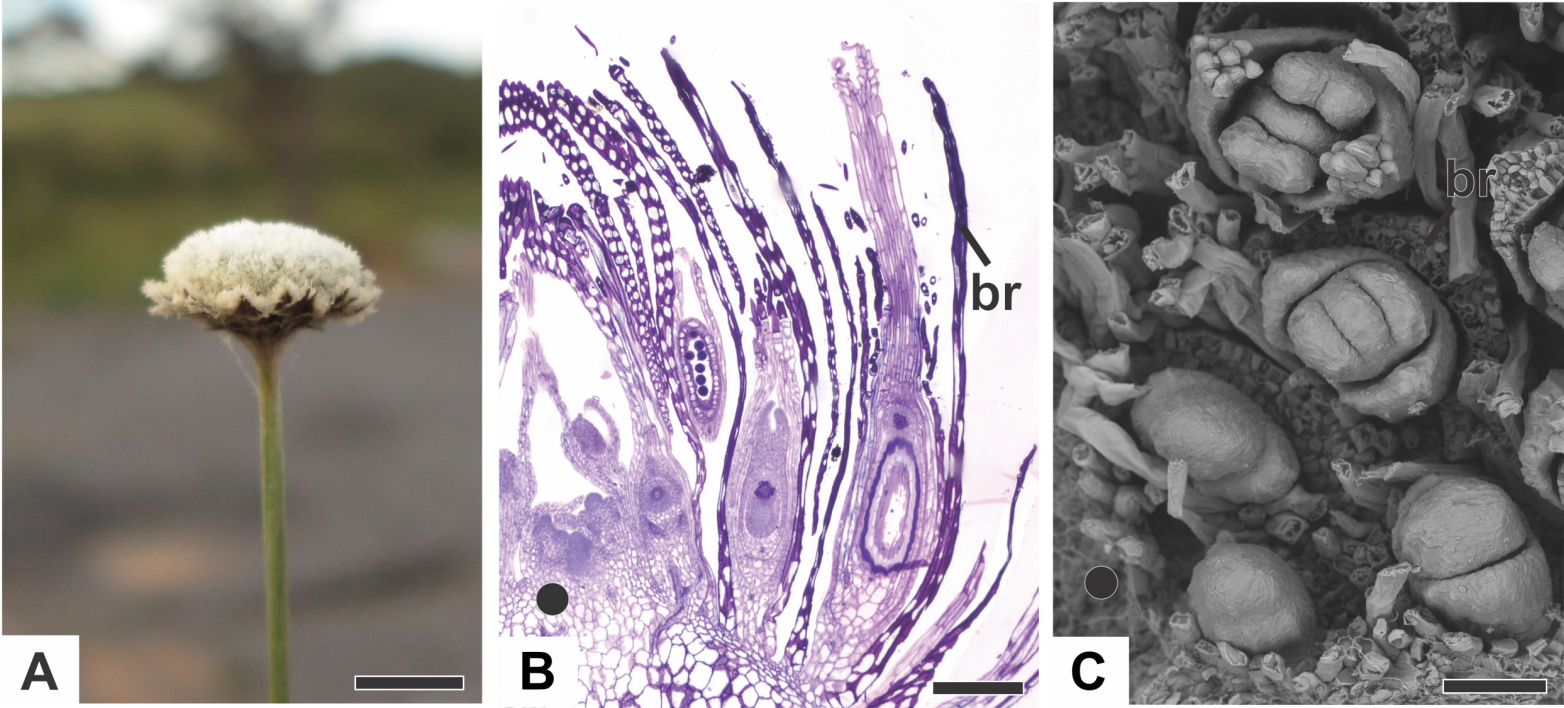
Aspects of the capitulum of dimerous species of *Paepalanthus*. (A) General aspect of the capitulum of *P. elongatus*. (B) Detail of a longitudinal section (LS) of the capitulum of *P. scleranthus* (light microscopy—LM). (C) Young flowers, with bracts removed, in the capitulum of *P. vaginatus* (Scanning Electron Microscopy—SEM). Labels: •, inflorescence centre; br, floral bract. Scale bars: A = 1.5 cm; B = 200 µm; C = 60 µm.

In all the dimerous *Paepalanthus* species studied, the flowers are born in bisexual capitula (Fig. 1A). The flowers are unisexual and subtended by an abaxial floral bract (Fig. 1B). The capitula exhibit centripetal development, so that the oldest flowers occur at the periphery and the youngest in the centre (Figs. 1B and 1C). Trimerous flowers are extremely rare in these taxa, so these species are considered exclusively dimerous.

### Organography of staminate flower

**Figure 2:**
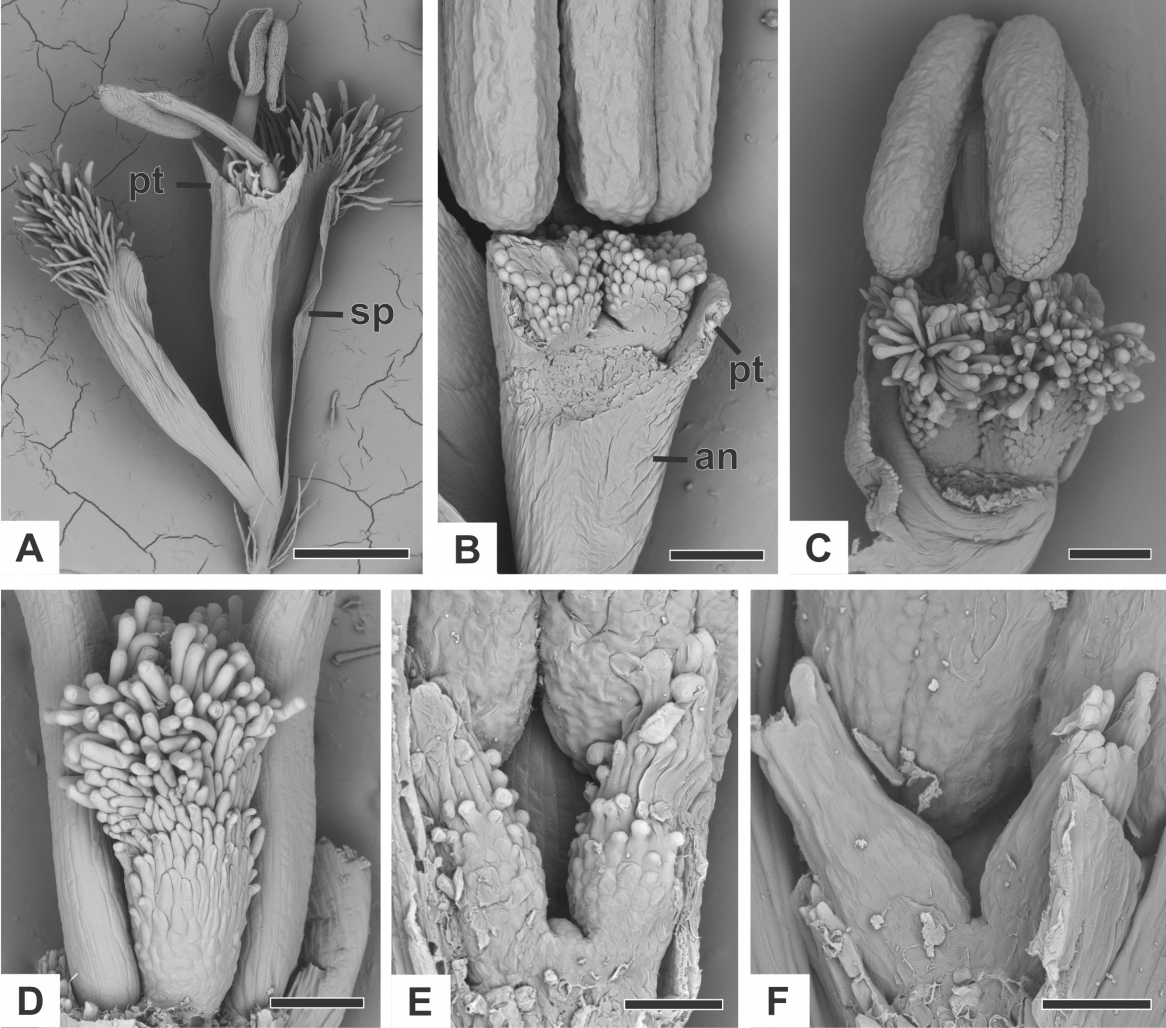
Organography of staminate flowers of dimerous species of *Paepalanthus* (SEM). (A) Staminate flower of *P. elongatus*. (B–C) Successive stages of mature flowers of *P. urbanianus* with corolla opened and one stamen removed. (D–F) Details of carpellodes of *P.chiquitensis* (D), *P. flaccidus* (E) and *P. scleranthus* (F). Labels: an, anthophore; pt, petal; sp, sepal. Scale bars: A = 300 µm; B = 90 µm; C, D = 150 µm; E = 60 µm; F = 40 µm.

The staminate flower of dimerous species of *Paepalanthus* are pedicellate and have two perianth whorls (Fig. 2A). The calyx consists of two free sepals (Fig. 2A). An anthopore subtends the corolla, the androecium and the modified gynoecium (Fig. 2B). In all studied species, the corolla is gamopetalous and consists of two fused petals, each one with a prominent apex (Fig. 2A). The androecium is haplostemonous and consists of two stamens with bithecous tetrasporangiate anthers (Figs. 2A and 2C). The gynoecium is modified into two nectariferous structures and a central bulge (Figs. 2B–2F). The nectariferous structures have long papillae in *P. chiquitensis* (Fig. 2D), *P. cordatus*, *P. elongatus*, *P. flaccidus* (Fig. 2D), *P. urbanianus* (Fig. 2E) and *P. vaginatus*. In *P. echinoides* and *P. scleranthus* (Fig. 2E), those structures have less prominent papillae. The nectariferous structures have a globular shape in *P. chiquitensis* (Fig. 2D), *P. cordatus*, *P. elongatus*, *P. urbanianus* (Fig. 2C) and *P. vaginatus*, whereas in *P. echinoides*, *P. flaccidus* (Fig. 2E) and *P. scleranthus* (Fig. 2E), they have a clavate shape.

### Organography of pistillate flower

**Figure 3:**
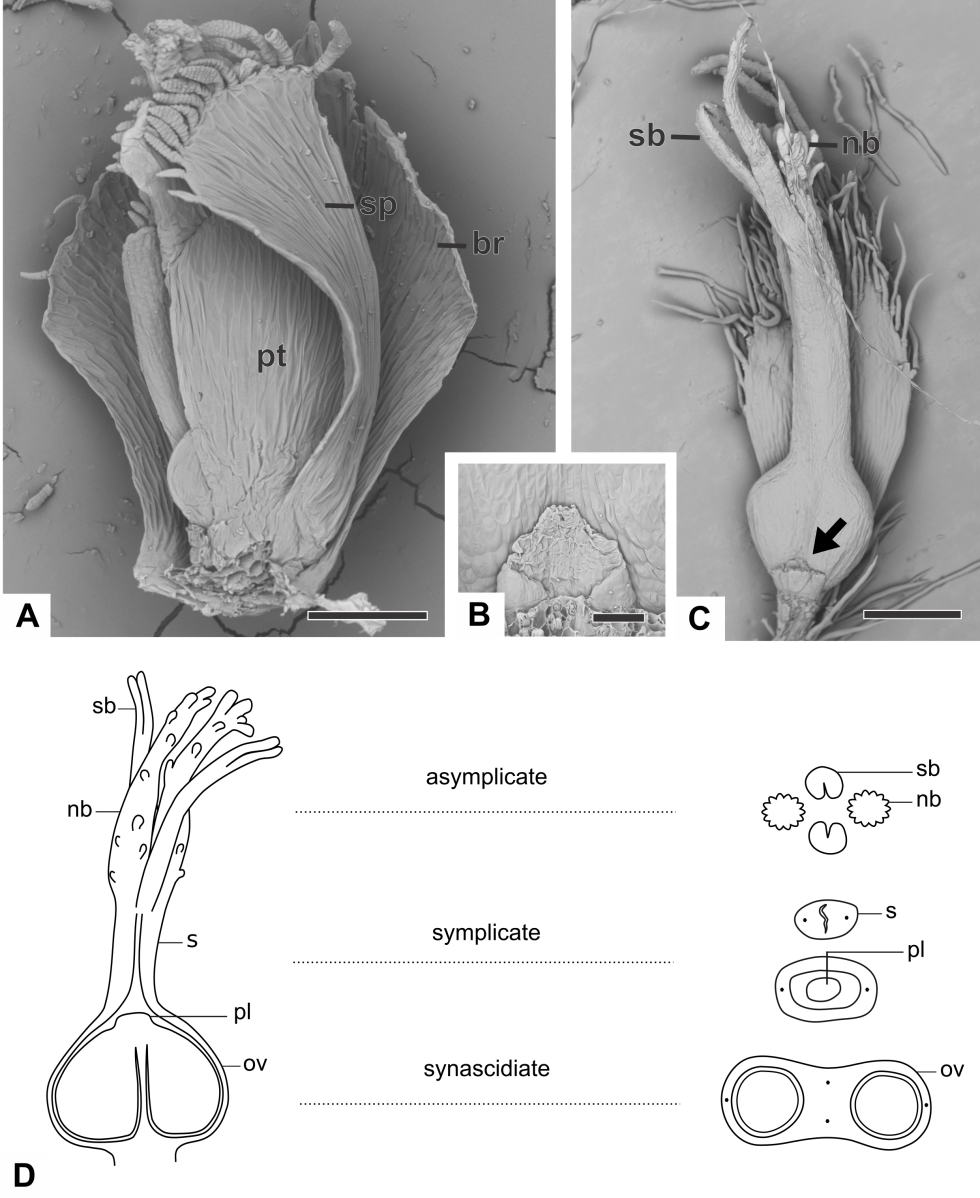
Organography of pistillate flowers of dimerous species of *Paepalanthus*. (A) Flower of *P. echinoides* with one sepal removed (SEM). (B) Detail of the staminode of *P. elongatus* (SEM). (C) Gynoecium of *P. elongatus* showing both nectariferous and stigmatic branches. (D) Schematic representation of the gynoecium of *P. flaccidus* with cross-sections showing its zones. Labels: arrow, staminode; br, floral bract; nb, nectariferous branch; ov, ovary; pl, placenta; pt, petal; s, style; sb, stigmatic branch; sp, sepal. Scale bars: A = 150 µm; B = 40 µm; C = 400 µm.

The pistillate flower of dimerous species of *Paepalanthus* are pedicellate and have perianth with two whorls (Fig. 3A). The calyx consists of two free sepals and the corolla consists of two free petals. The androecium is reduced to two scale-like staminodes (Fig. 3B). The gynoecium in all species is superior, eusynascidiate, with a bilocular ovary (Figs. 3C–3E). It consists of three distinct zones: a proximal one, synascidiate, a median symplicate, and a distal one, asynascidiate (Fig. 3E).

**Figure 4:**
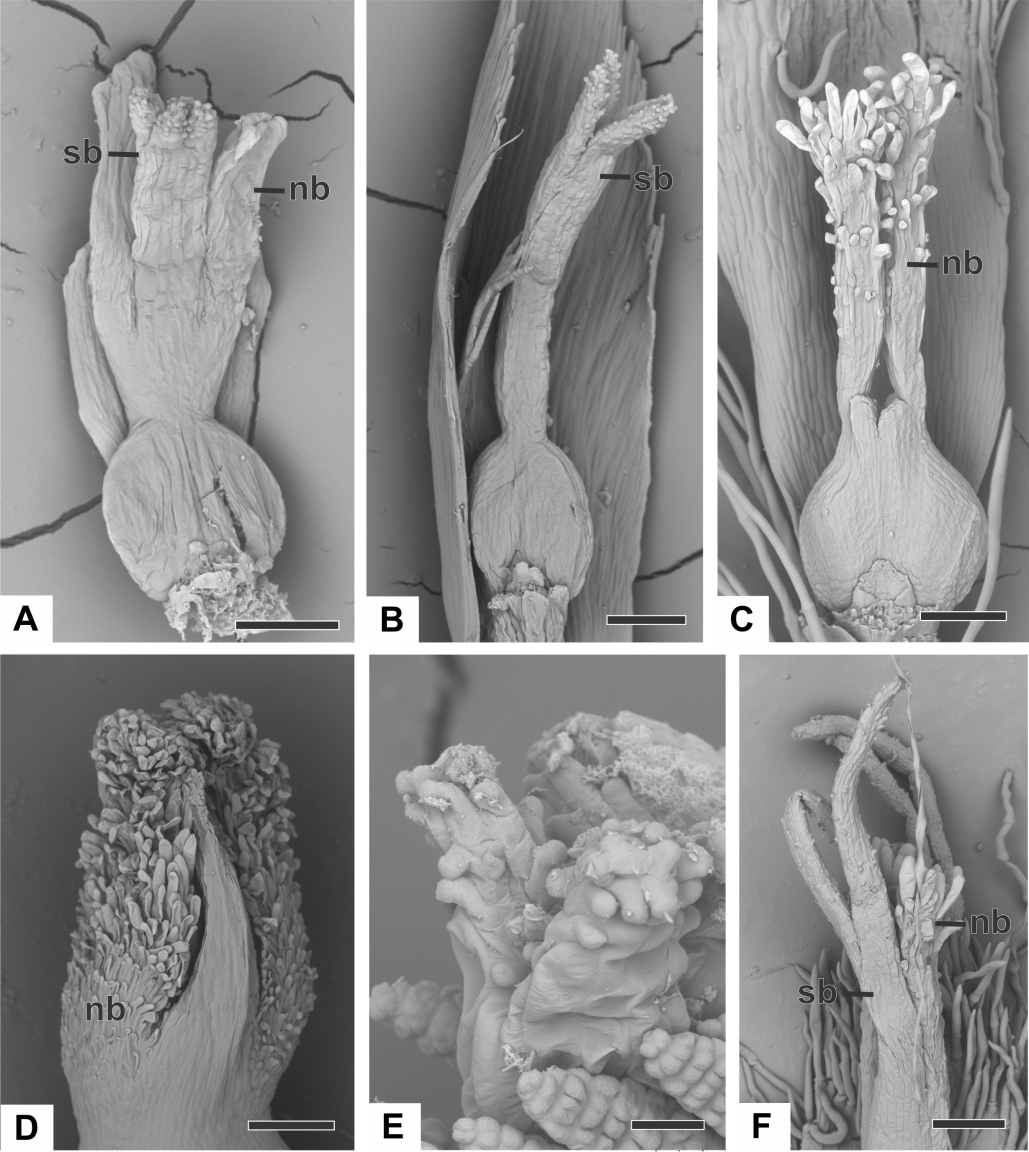
Organography of pistillate flowers of dimerous species of *Paepalanthus* (SEM). (A) Gynoecium of *P. echinoides* with both nectariferous and stigmatic branches. (B) Gynoecium of *P. scleranthus* showing stigmatic branches. (C) Gynoecium of *P. elongatus* with aborted stigmatic branches and developed nectariferous branches. (D) Detail of the apical region of the gynoecium of *P. cordatus*. (E) Detail of the stigma of *P. echinoides*. (F) Detail of both nectariferous and stigmatic branches of *P. elongatus*. Labels: nb, nectariferous branch; sb, stigmatic branch. Scale bars: A, B = 100 µm; C = 150 µm; D, F = 200 µm; E = 15 µm.

With respect to its basic morphology, the studied species have three distinct patterns of pistillate flowers. In the first pattern, flowers have a gynoecium with two nectariferous branches and two long stigmatic branches inserted at the same point on a short style, as observed in *P. cordatus*, *P. elongatus* (Fig. 3C) and *P. flaccidus*. In the second pattern, flowers have a gynoecium with two nectariferous branches inserted at the low median part of a long style and two short stigmatic branches inserted at its end point (Fig. 4A). This pattern was found only in mature flowers of *P. echinoides* (Fig. 4A). In the third pattern, flowers have a gynoecium without nectariferous branches and with two short stigmatic branches inserted at the end on a long style (Fig. 4B). This latter pattern was found only in mature flowers of *P. scleranthus* (Fig. 4B). In *P. chiquitensis*, *P. urbanianus* and *P. vaginatus*, mature pistillate flowers were not found in the collected capitula. In *P. elongatus* (Fig. 4C), some sterile flowers were also found, in which stigmatic branches do not arise, but nectariferous branches (nb) are completely developed and appear to be functional.

In almost all studied species, the nectariferous branches have long papillae (Figs. 4C and 4D), except in *P. echinoides* (Fig. 4A), whose branches lack papillae; and in *P. scleranthus* (Fig. 4B), which lacks nectariferous branches. *Paepalanthus echinoides* (Fig. 4A), *P. elongatus* (Fig. 3C) and *P. flaccidus* have nectariferous branches with a thin base, whereas in *P. cordatus* (Fig. 4D), they have a broad base.

Stigmas in all studied species are bifid, with papillose epidermis (Figs. 4E and 4F). The stigmas are bifid only at the end of the stigmatic branches in *P. echinoides* (Figs. 4B and 4E), *P. cordatus*, *P. flaccidus* and *P. scleranthus* (Fig. 4B), whereas in *P. elongatus* (Fig. 4F), the stigma is bifid for half of their length. In *P. echinoides* (Fig. 4A) and *P. scleranthus* (Fig. 4B), the two bifid stigmas are held close together in the style apex.

### Ontogeny

**Figure 5:**
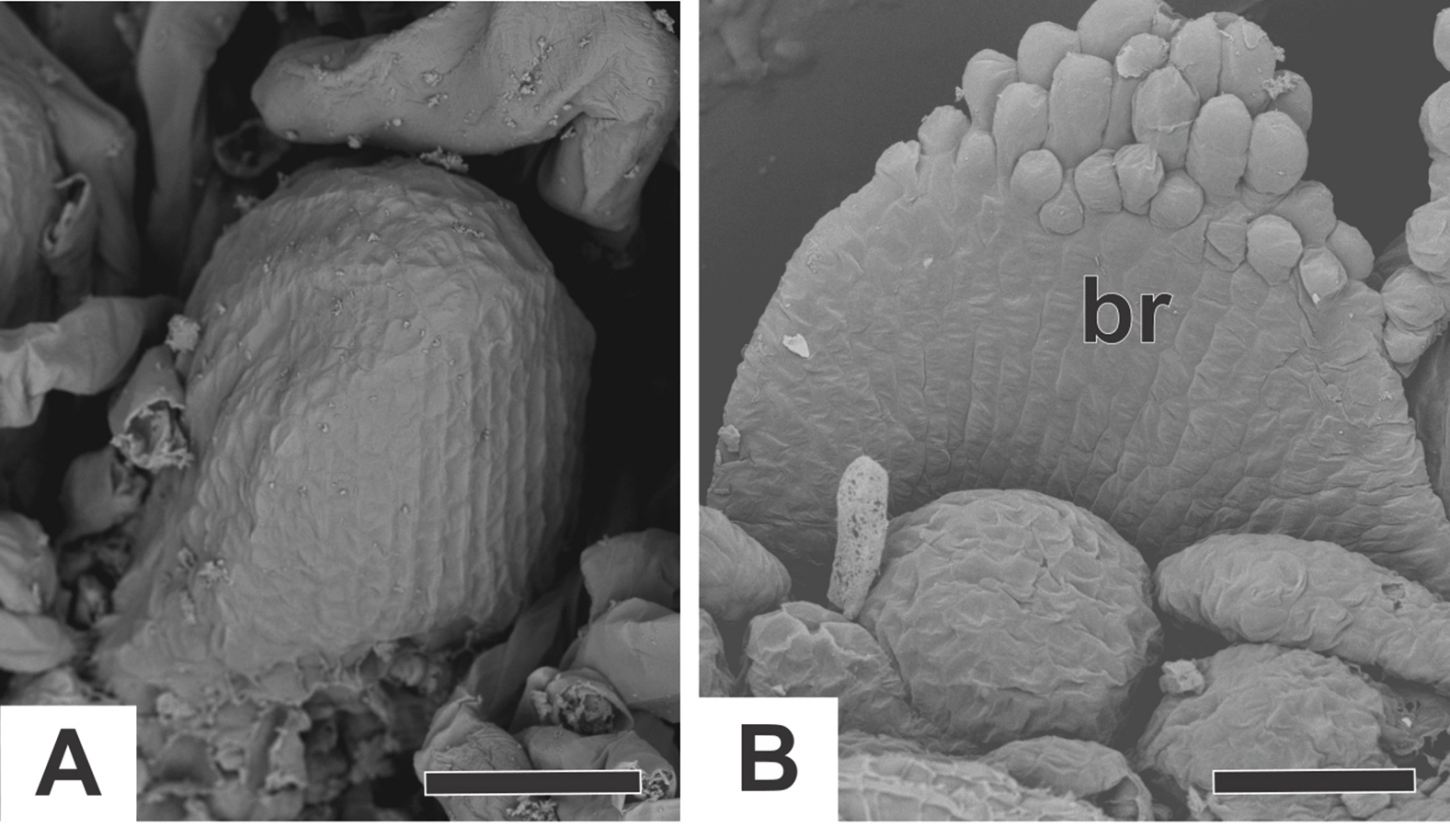
Floral primordia of dimerous species of *Paepalanthus* (SEM). (A) Floral primordium of *P. chiquitensis*. (B) Floral primordium of *P. flaccidus*. Label: br, floral bract. Scale bars: A, B = 30 µm.

The floral development of both sexes begins with a dome-shaped primordium protected by the floral bract (Figs. 5A and 5B). The developmental process consists of the following stages: the emergence of floral parts of each whorl from the floral primordium; the sex differentiation of both staminate and pistillate flowers; and the maturation of all floral parts until anthesis. In the initial floral developmental stages, there is no distinction between staminate and pistillate flowers. The flower development is centripetal; hence, the results are presented in the order in which the floral parts emerge from the primordium.

### Ontogeny of staminate flower

**Figure 6:**
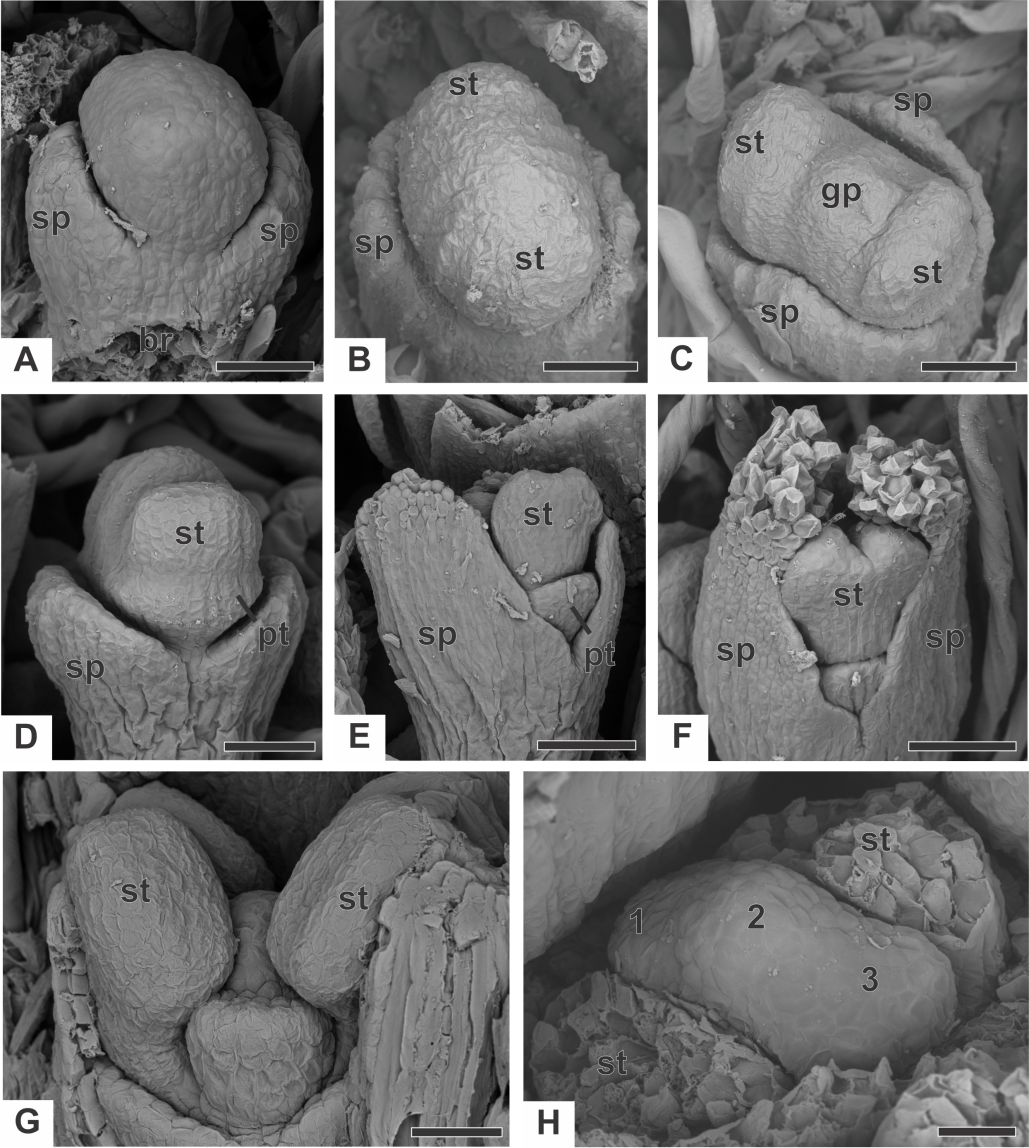
Early developmental stages of staminate flowers of dimerous species of *Paepalanthus* (SEM). (A) Floral primordium of *P. chiquitensis* (floral bract removed) showing sepals. (B, C) Floral primordia of *P. urbanianus* at successive stages showing stamen and gynoecium initiation. (D, E) Developing flowers of *P. cordatus* showing late-developing petals. (F) Developing flower of *P. chiquitensis*. (G) Developing flower of *P. flaccidus* with sepals removed. (H) Developing flower of *P. vaginatus* with stamens removed to show the bulges (1, 2 and 3) in the gynoecium. Labels: br, floral bract; gp, gynoecium primordium; pt, petal; sp, sepal; st, stamen. Scale bars: A, B = 30 µm; C = 50 µm; D = 60 µm; E, F = 80 µm; G, H = 20 µm.

In staminate flowers of dimerous species of *Paepalanthus*, the first whorl to emerge from the floral primordium is the calyx, which is formed by two sepal primordia (sp) alternate with the floral bract (Fig. 6A). Development initiates simultaneously for both lateral sepals. The corolla develops late, and the outer stamen whorl (which would be expected to be opposite the sepals) is absent during the whole flower ontogeny. Therefore, sepal initiation is followed by the appearance of the common petal-stamen primordia alternate with the calyx (Fig. 6B). The gynoecium primordium appears in the central region of the floral primordium (Fig. 6C). Two petal and stamen primordia are initiated shortly after, by division of the common petal-stamen primordia (Figs. 6D and 6E). From the beginning of floral development, the sepals protect the young flower (Figs. 6E and 6F). During this process, the anther thecae differentiate within the stamen primordia (Figs. 6E and 6F). Each stamen primordium differentiates into a filament and anther (Fig. 6G). The gynoecium primordium divides into three bulges in a line (two lateral and one central), whose lateral ones alternate with the stamens (Fig. 6H).

**Figure 7:**
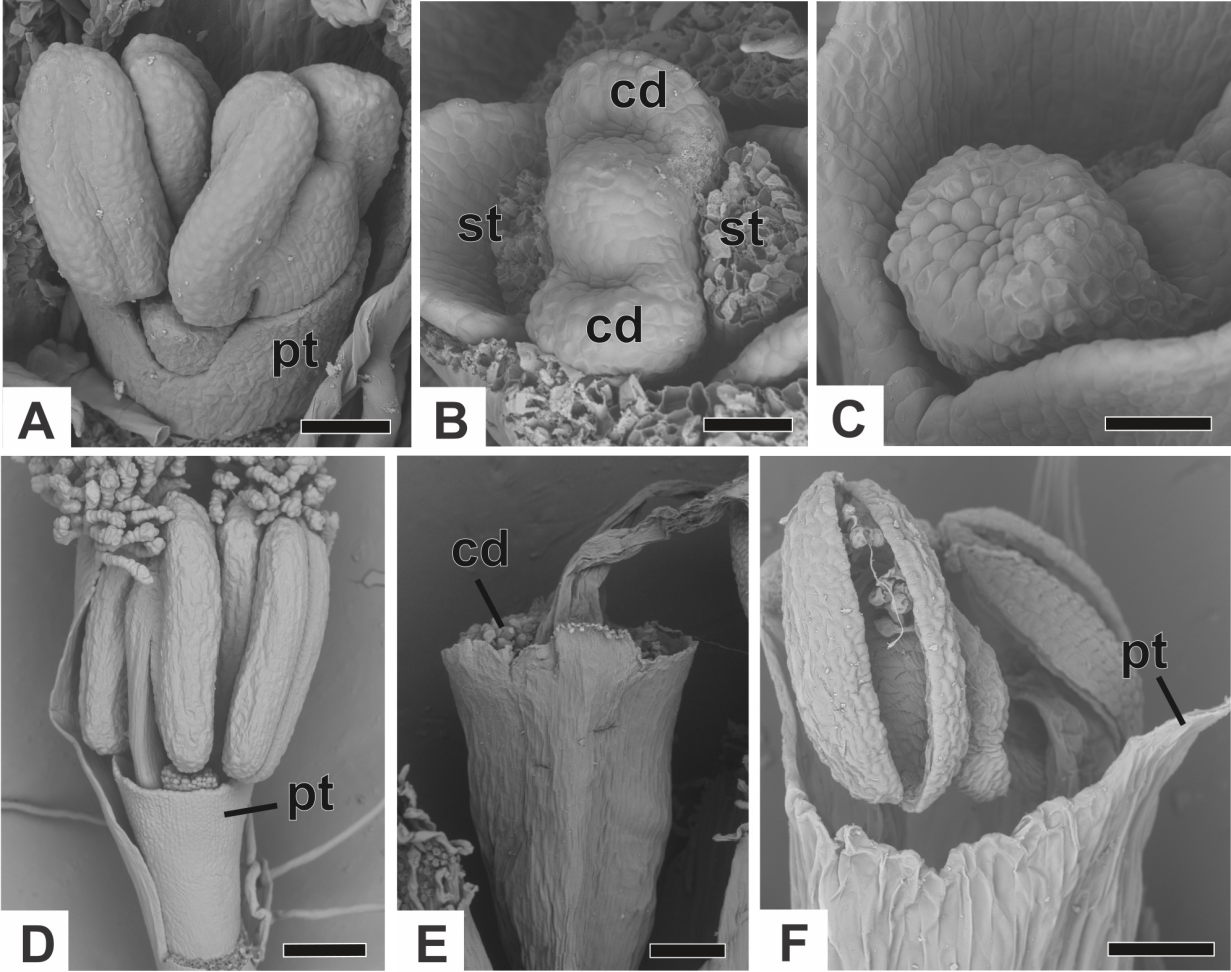
Successive developmental stages of staminate flowers of dimerous species of *Paepalanthus* (SEM). (A) Developing flower of *P. chiquitensis* with sepals removed showing initiation of the corolla tube. (B) Detail of a developing flower of *P. chiquitensis* with sepals and stamens removed to show early development of carpellodes. (C) Detail of a developing carpellode of *P. chiquitensis*. (D) Immature flower of *P. urbanianus* with sepal removed. (E) Flower of *P. cordatus* at anthesis, with carpellodes at the same level of the corolla tube opening. (F) Flower of *P. scleranthus* at anthesis. Labels: cd, carpellode; pt, petal; st, stamen. Scale bars: A, C = 40 µm; B = 20 µm; D, F = 60 µm; E = 100 µm.

The corolla consists of two separate petals, which fuse together later in development (Fig. 7A). The lateral prominences in the gynoecium primordium differentiate into lobes, while the central prominence remains as an undifferentiated dome (Fig. 7B). The lobes correspond to the nectariferous carpel walls, or carpellodes (cd), and each one soon acquires a cylindrical shape and papillate surface (Fig. 7C). Stamens and nectariferous carpellodes develop and the corolla tube elongates (Fig. 7D).

**Figure 8:**
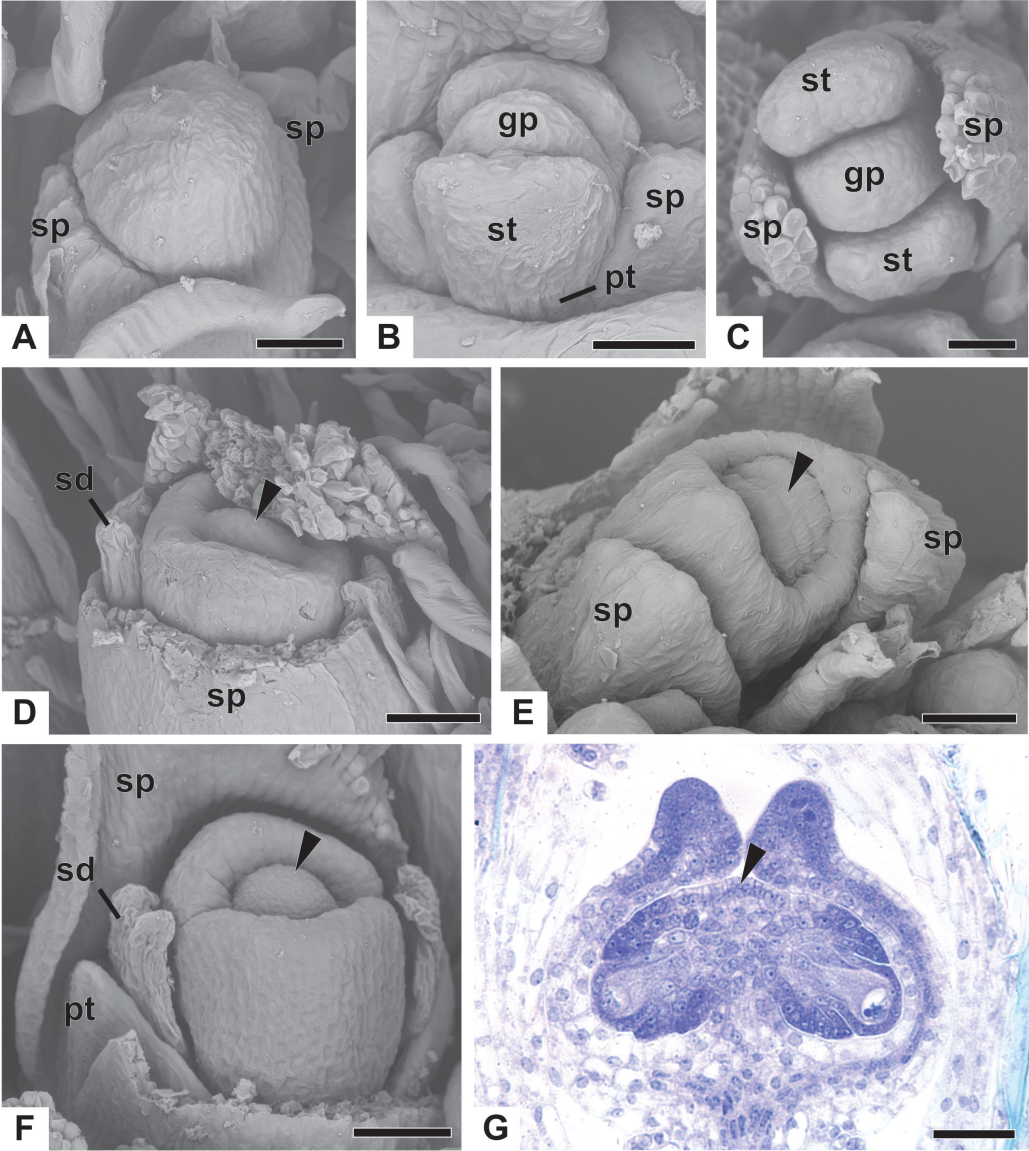
Early developmental stages of pistillate flowers of dimerous species of *Paepalanthus*. (A) Floral primordium of *P. chiquitensis* showing initiation of sepals (SEM). (B) Floral primordium of *P. echinoides* showing sepals, stamens, gynoecium and late-developing petals (SEM). (C) Developing flower of *P. chiquitensis* in frontal view (SEM). (D) Developing flower of *P. chiquitensis* with one sepal removed and detail of the ovarian septum (arrowhead) separating the locules (SEM). (E) Developing flower of *P. scleranthus* with asymmetric sepals (SEM). (F) Developing flower of *P. cordatus* with one sepal removed showing petal, staminodia and gynoecium (SEM). (G) Longitudinal section of young gynoecium of *P. echinoides* showing ovules and ovarian septum (LM). Labels: arrowhead, ovarian septum; gp, gynoecium primordium; pt, petal; sd, staminode; sp, sepal; st, stamen. Scale bars: A, C = 40 µm; B, E = 20 µm; D, F = 60 µm; G = 250 µm.

At anthesis, the filaments project the anthers above the petals, and the anthers have latrorse dehiscence (Figs. 7E and 7F). Nectariferous carpellodes are located at the same height as, or exposed above, the corolla tube in *P. chiquitensis*, *P. cordatus* (Fig. 7E), *P. elongatus*, *P. flaccidus*, *P. urbanianus* and* P. vaginatus*. In *P. echinoides* and *P. scleranthus* (Fig. 7F), carpellodes remain protected below the corolla opening.

### Ontogeny of pistillate flower

Early development of the pistillate flowers of dimerous *Paepalanthus* is similar to that observed in staminate flowers. The calyx is the first whorl to appear, with two sepals that alternate with the floral bract, emerging simultaneously from the floral primordium (Fig. 8A). Calyx appearance is followed by the emergence of the common petal-stamen primordia, alternate with the sepals; then the gynoecium primordium in the central region of the floral primordium; and then by a split in the common petal-stamen primordia, resulting in both petal and stamen primordia (opposite to each other) (Figs. 8B and 8C). The pistillate flower is recognizable by the gynoecium differentiation, which is marked by the congenital fusion of the lateral bulges of gynoecium primordium (which correspond to the carpel walls) and, consequently, by the initiation of the synascidiate zone of the gynoecium (Figs. 8D and 8E). The central bulge of the gynoecium primordium corresponds to the septum (arrowhead) and divides the two locules, which are opposite the sepals (Fig. 8D). Early stages of carpel development were found only in *P. chiquitensis* (Fig. 8D), *P. echinoides* and *P. scleranthus* (Fig. 8E). In *P. scleranthus* (Fig. 8E), some young flowers have sepals in a slightly asymmetric position, not exactly alternate to the floral bract.

After ovary differentiation, the sepals protect the young flower, and petals develop (Fig. 8F). During ovary development, the carpels form a ring around the septum, forming the symplicate zone (Fig. 8F). The stamens stop their development, becoming scale-like staminodes (sd) (Fig. 8F). The ovary septum raises the placenta, and the ovules become pendulous (Fig. 8G).

**Figure 9:**
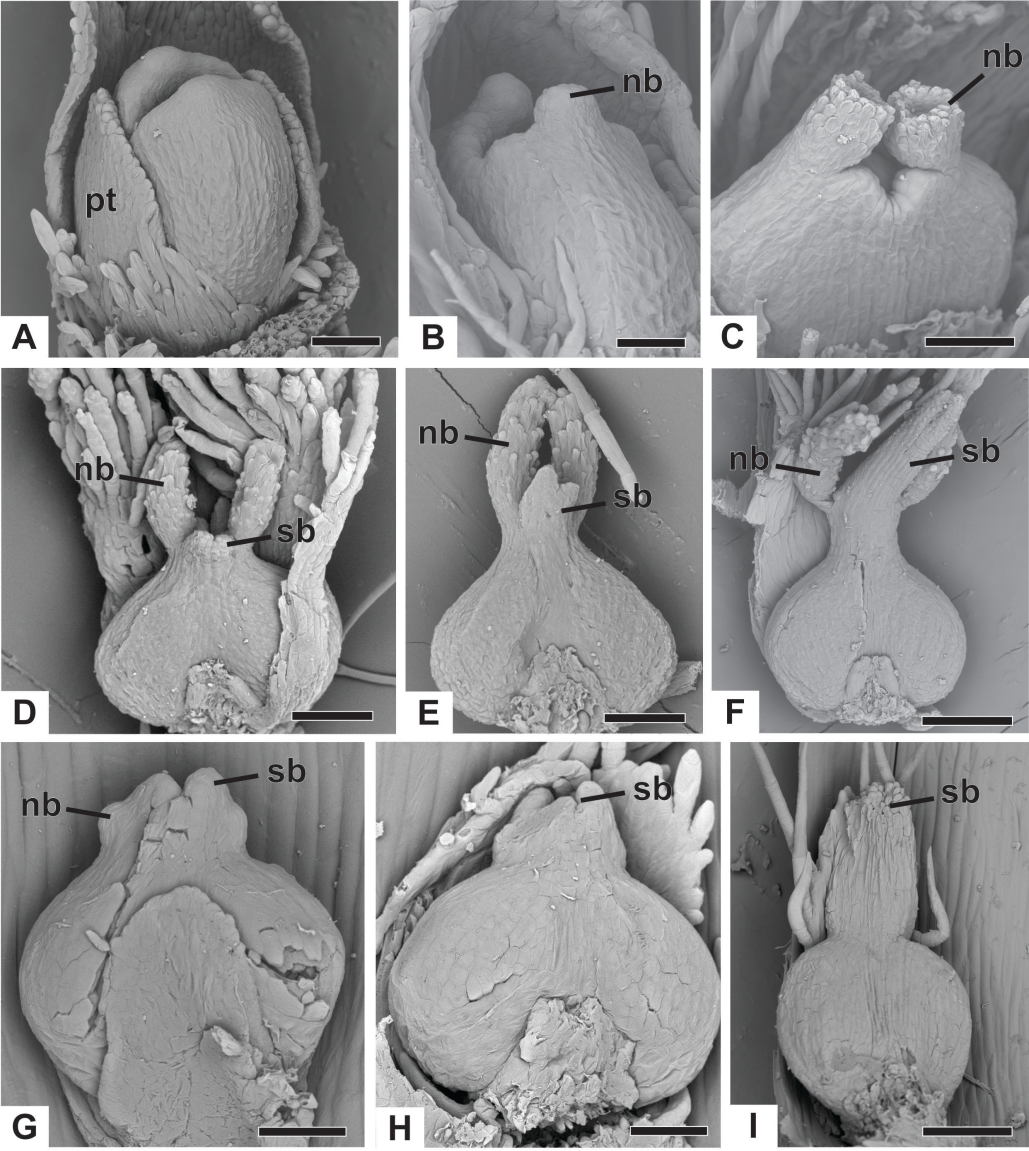
Successive developmental stages of pistillate flowers of dimerous species of *Paepalanthus* (SEM). (A) Developing flower of *P. urbanianus* with one sepal removed. (B) Developing gynoecium of *P. flaccidus* with the inception of nectariferous branches at the carpel apices. (C) Developing gynoecium of *P. vaginatus* showing nectariferous branches. (D–F) Developing gynoecia of *P. flaccidus* with elongation of the stylar branches at the carpel margins. (G) Developing gynoecium of *P. scleranthus* with early development of both nectariferous and stigmatic branches. (H) Gynoecium of *P. scleranthus* with inception of stigmatic branches. (I) Gynoecium of *P. scleranthus* with fused stigmatic branches. Labels: nb, nectariferous branch; pt, petal; sb, stigmatic branch. Scale bars: A, C = 60 µm; B = 30 µm; D, I = 120 µm; E = 90 µm; F = 200 µm; G, H = 40 µm.

The apical portions of the carpels differentiate progressively into cylindrical structures with papillose epidermis (Figs. 9A–9C). These structures appear in carinal position and correspond to the nectariferous branches (nb) of the gynoecium. The stigmatic branches are formed later in the commissural position by the apical growth of the tissue on the boundary between the carpels (sb) (Figs. 9D–9F). During the formation of the stigmatic branches, the nectariferous branches elongate (Figs. 9D–9F). The style is formed by the intercalary growth of the region between the gynoecium branches and the ovary (Figs. 9E and 9F). The stigma is formed by the incomplete fusion of the adjacent carpel margins (Figs. 9D–9F).

In *P. scleranthus*, the primordia of the nectariferous branches may arise in some flowers (Fig. 9G), but in general they do not develop, becoming superficially absent during the gynoecium development (Figs. 9H and 9I). In this species, the style is formed by intercalary growth of the region between the ovary and the short stigmatic branches, resulting in a long columnar structure (Fig. 9I). In *P. echinoides*, the developmental stages associated with carpel closure and branch formation were not observed due to the absence of these stages in the collected capitula.

**Figure 10:**
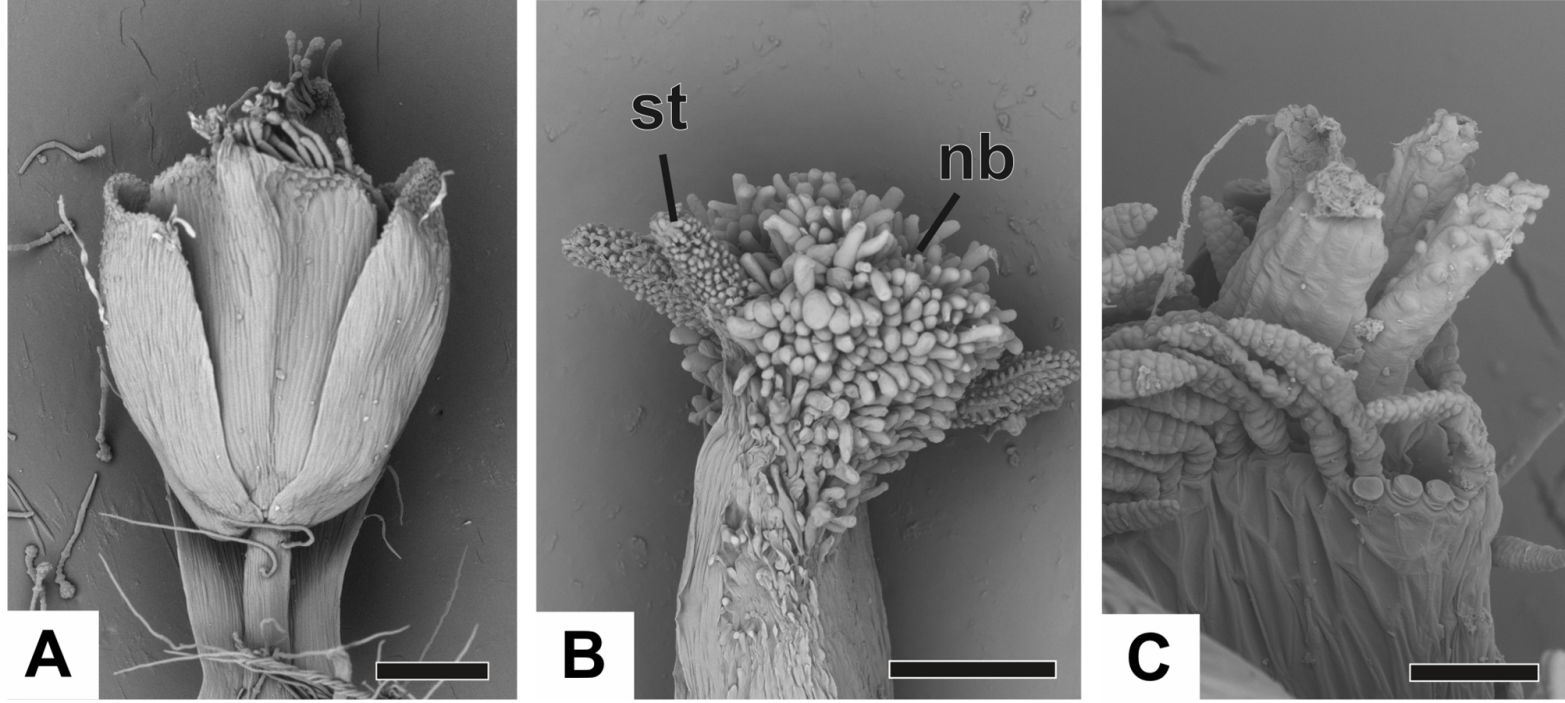
Anthesis of pistillate flowers of dimerous species of *Paepalanthus* (SEM). (A) Pistillate flower of *P. flaccidus*. (B) Detail of both stigmatic and nectariferous branches in *P. cordatus*. (C) Detail of the stigma of *P. echinoides*. Labels: nb, nectariferous branch; st, stigma. Scale bars: A, B = 300 µm; C = 60 µm.

At female anthesis in all species, sepals reach the flower apex and petals develop, almost reaching the height of the sepals (Fig. 10A). Stigmatic and nectariferous branches elongate and are exposed above the sterile floral parts, along with the stigma surface (s) (Figs. 10B and 10C).

### Vascularization of staminate flower

**Figure 11:**
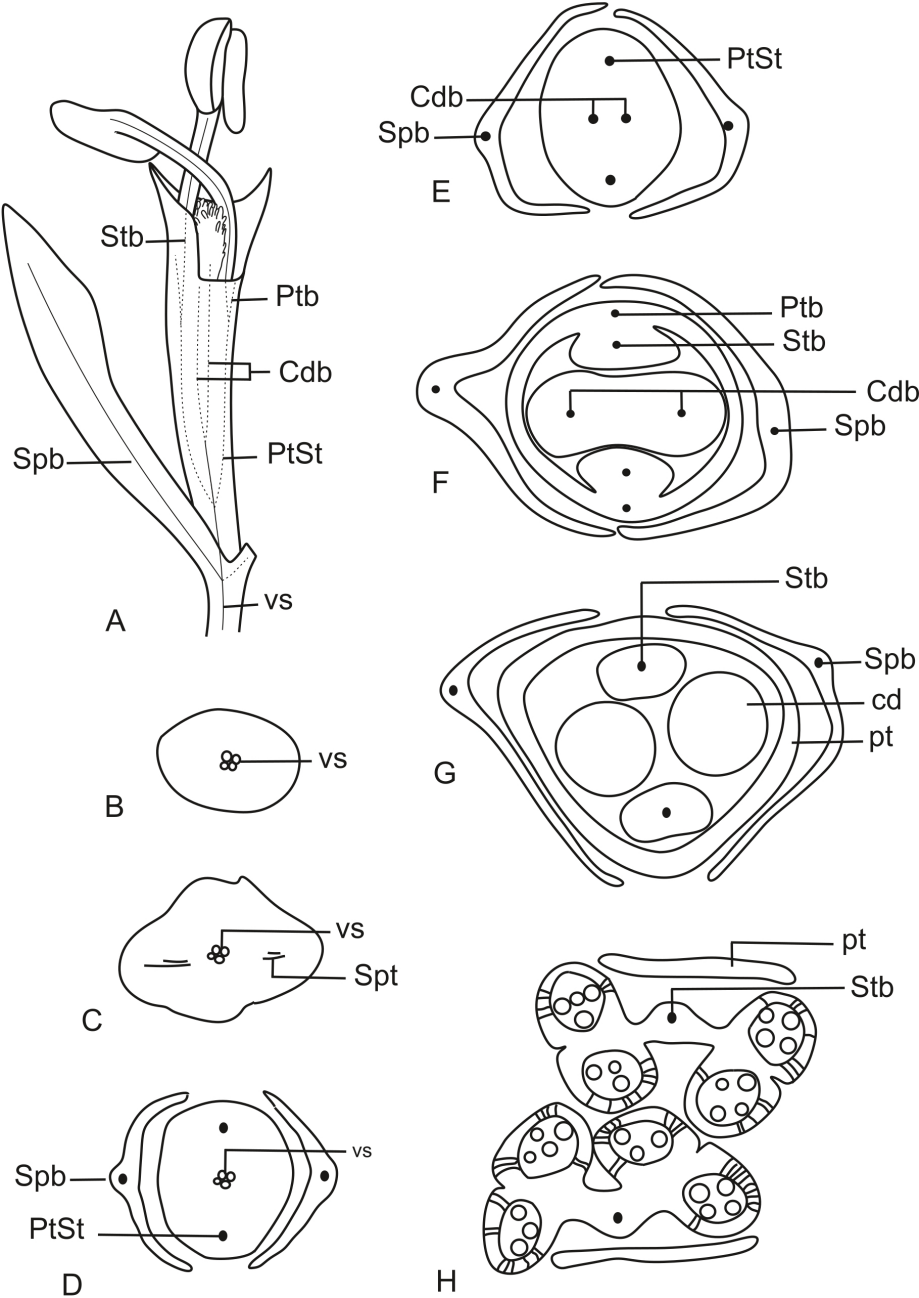
Schematic representation of the vascularization of staminate flowers of *P. elongatus*. (A) Diagram of an entire mature flower with one sepal removed and detail of its vascularization. (B–H) Diagrams of cross-sections of the flower from the pedicel (B) up to the apical region of the flower (H). Labels: cd, carpellode; Cdb, carpellode vascular bundle; pt, petal; Ptb, petal vascular bundle; PtSt, petal-stamen complex; Spb, sepal vascular bundle; Stb, stamen vascular bundle; vs, vascular strand.

In Fig. 11A, we present the schematic representation of a staminate flower of a dimerous *Paepalanthus*, with details of its vascularization. Representations of cross-sections of the flower are also illustrated (Figs. 11B–11H), from the pedicel (Fig. 11B) to the floral apex (Fig. 11H).

At the flower base, the pedicel is supplied by a single vascular strand (vs) (Fig. 11B). Two vascular traces, corresponding to the sepal traces (Spt), diverge from the vascular strand (Fig. 11C). Above the sepal insertions, two vascular bundles diverge alternate to the sepals (Fig. 11D). These bundles correspond to the petal-stamen complex (PtSt) (Fig. 11D). The remaining central vascular strand divides into two vascular bundles opposite to the sepals, which vascularize the carpellodes (Cdb) (Fig. 11E). Each of the petal-stamen complexes finally divides into two bundles opposite to each other; these are the vascular bundles of the petals (Ptb) and stamens (Stb) (Figs. 11F–11H).

**Figure 12:**
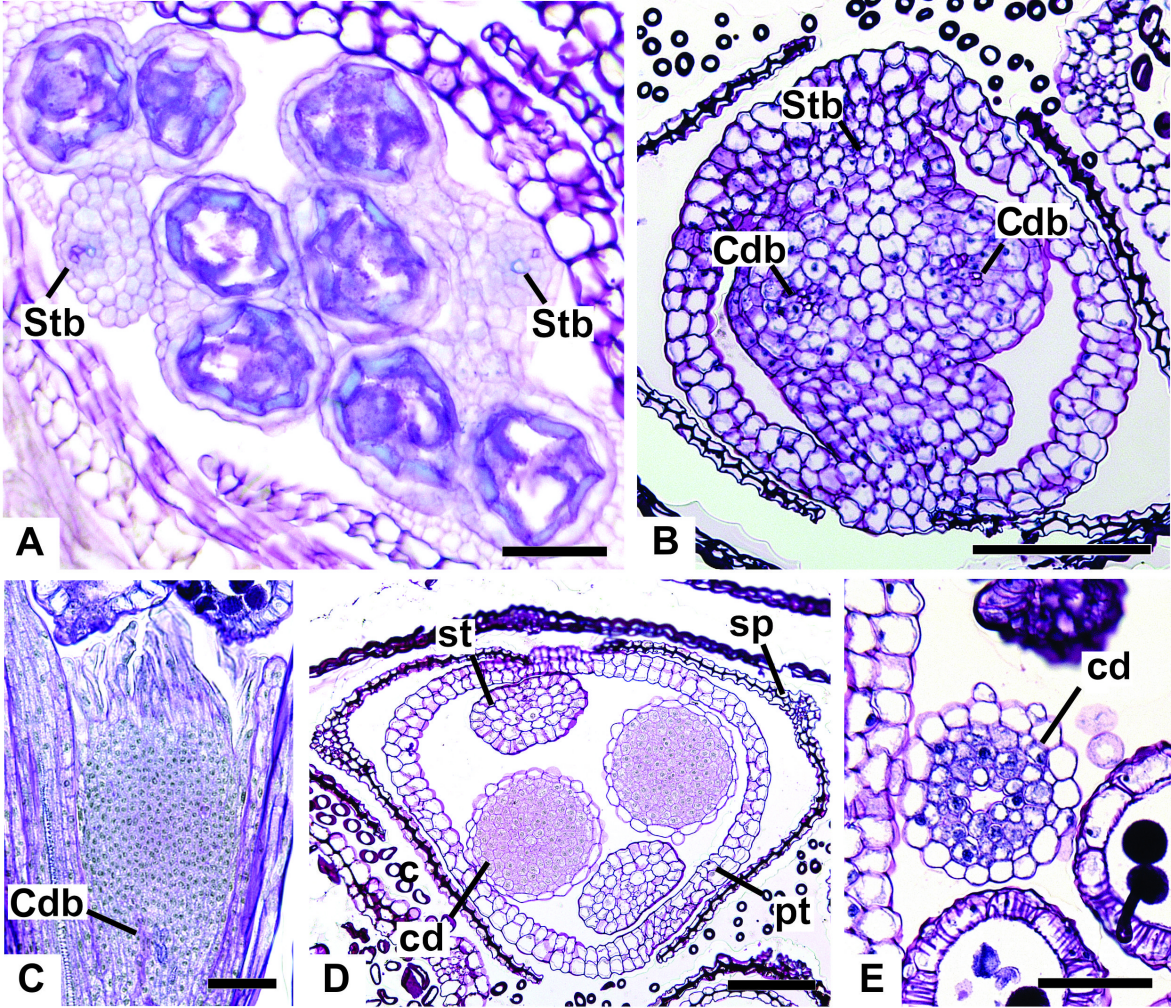
Details of staminate flowers of dimerous species of *Paepalanthus* (LM). (A) Developing anthers of *P. scleranthus*, in cross-section (CS). (B) Flower of *P. flaccidus* at the base of carpellodes, in CS. (C) Detail of nectariferous carpellodes of *P. chiquitensis*, in longitudinal section (LS). (D) Flower of *P. flaccidus* in the median region of carpellodes, in CS. (E) Detail of the apical region of carpellodes of *P. flaccidus*, in CS. Labels: cd, nectariferous carpellode; Cdb, carpellode vascular bundle; pt, petal; sp, sepal; st, stamen; Stb, stamen vascular bundle. Scale bars: A = 400 µm; B–E = 500 µm.

All floral parts are supplied by one single collateral bundle formed by 2–4 transport cells (Figs. 12A and 12B). However, the nectariferous carpellodes are supplied only at their base (Fig. 12C) and may have more transport cells (Fig. 12B). The cross-sections of the carpellodes clearly show that they are not vascularized along their entire extent (Figs. 12D and 12E).

### Vascularization of pistillate flower

**Figure 13:**
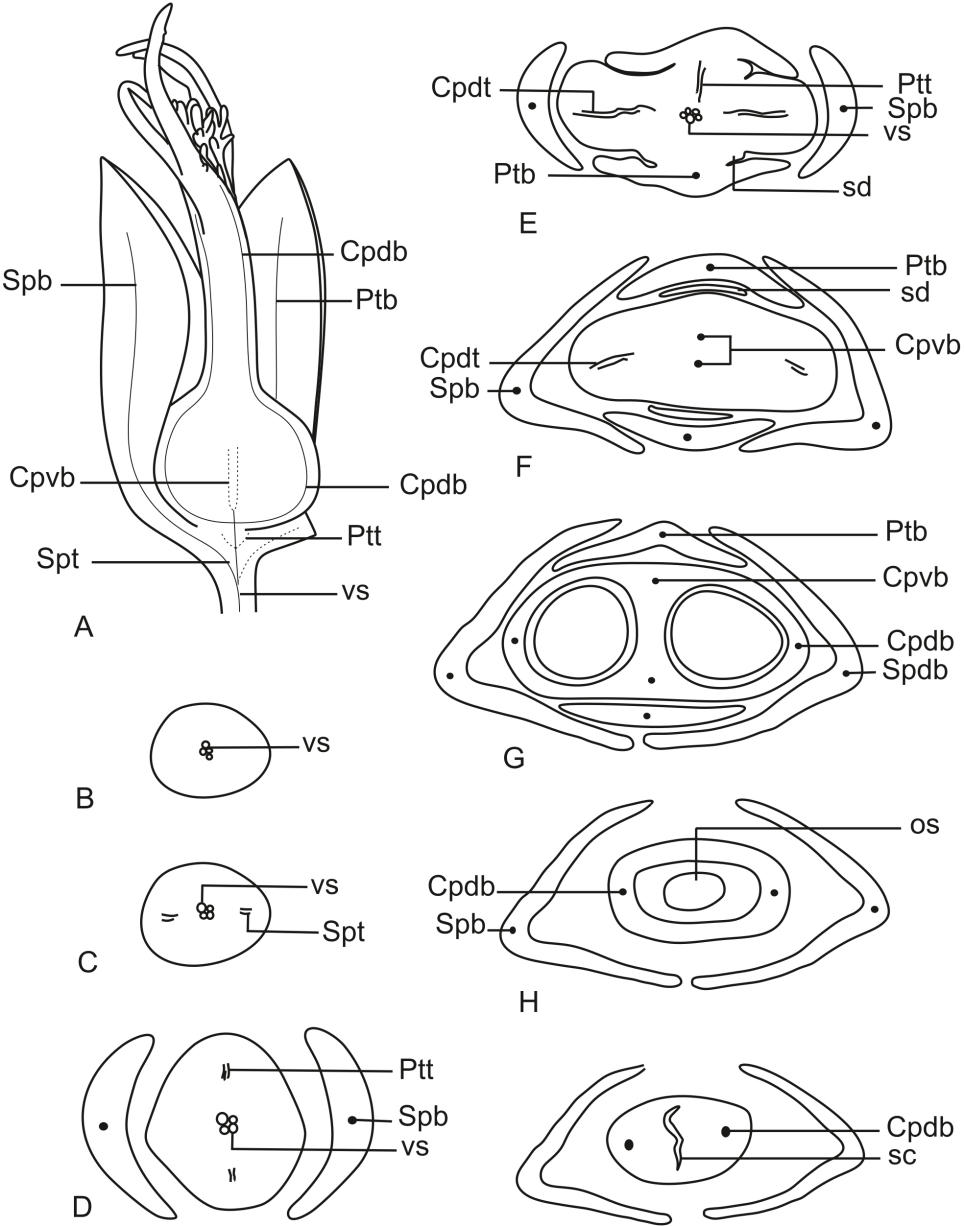
Schematic representation of the vascularization of pistillate flowers of *P. elongatus*. (A) Diagram of an entire mature flower with one sepal and one petal removed and detail of its vascularization. (B–I) Diagrams of cross-sections of pistillate flower from the pedicel (B) up to the style region (I). Label: Cpdb, carpel dorsal vascular bundle; Cpdt, carpel dorsal vascular trace; Cpvb, carpel ventral vascular bundle; Ptb, petal vascular bundle; Ptt, petal vascular trace; sc, stylar canal; sd, staminode; Spb, sepal vascular bundle; os, ovary septum; Spt, sepal vascular trace; vs, vascular strand.

**Figure 14:**
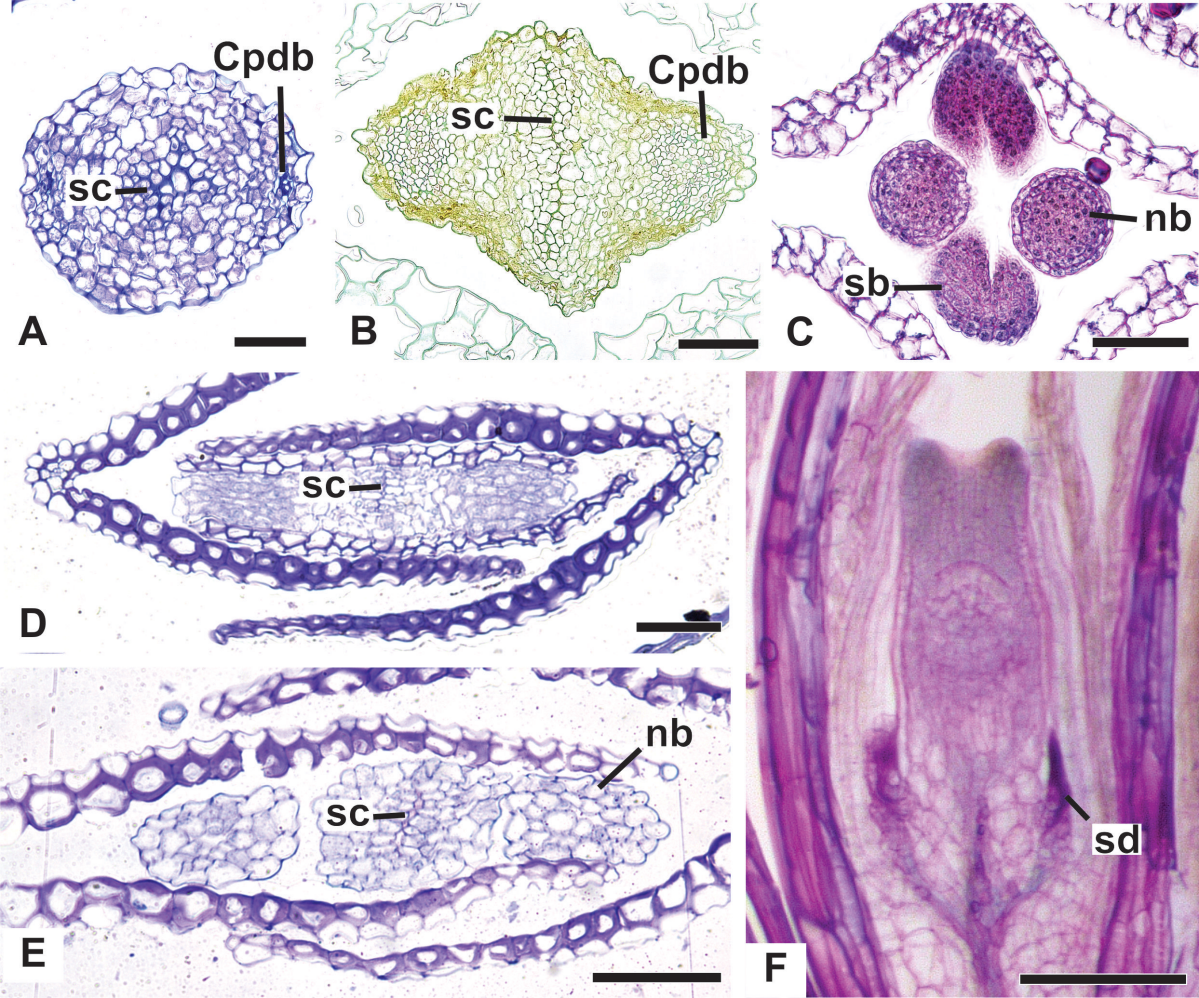
Details of pistillate flowers of dimerous species of *Paepalanthus* (LM). (A) Detail of the style of *P. elongatus*, in cross-section (CS). (B) Detail of the style of *P. chiquitensis* at the base of the nectariferous branches, in CS. (C) Detail of free nectariferous and stigmatic branches of *P. flaccidus*, in CS. (D) Flower of *P. echinoides* at the insertion of nectariferous branches, in CS. (E) Flower of *P. echinoides* at the median region of the gynoecium branches, in CS. (F) Detail of vascular bundles reaching the base of staminodes in *P. scleranthus*, in longitudinal section (LS). Labels: Cpdb, carpel dorsal vascular bundle; nb, nectariferous branch; sb, stigmatic branch; sc, stylar canal; sd, staminode. Scale bars: A–E = 50 µm; F = 100 µm; G = 80 µm.

In Fig. 13A, we present the schematic representation of a pistillate flower of dimerous *Paepalanthus*, with details of its vascularization. Representations of cross-sections are also illustrated (Figs. 13B–13I), from the region of the pedicel (Fig. 13B) to the style region (Fig. 13I). Details of the apical region of pistillate flowers are given in Fig. 14.

At the flower base, the pedicel is vascularized by a single vascular strand (vs) (Fig. 13B). From the central strand, two vascular traces diverge, corresponding to the sepal traces (Spt) (Fig. 13C). Two traces that alternate with the sepals diverge above sepal insertion and correspond to the petal traces (Ptt) (Figs. 13D and 13E).

Above the petal insertions, two vascular traces emerge opposite the sepals, equivalent to the dorsal carpellary traces (Cpdt) (Fig. 13E). Then, the central strand divides into two vascular bundles that alternate with the sepals, corresponding to the heterocarpellar ventral bundles of the ovary (Cpvb) (Figs. 13F and 13G). At the synascidiate zone of the gynoecium (Fig. 9G), the two ventral bundles are in the commissural position, and the dorsal bundles are opposite the sepals. In the symplicate zone of the gynoecium (Figs. 9H, 9I and 10A), only the two dorsal bundles of the carpels are present.

The nectariferous branches are vascularized only at their base (Fig. 14B). In this region, the dorsal bundles of the carpels may have more transport cells. The stigmatic branches are not vascularized in any of the studied species (Fig. 14C). In all species, the style has a stylar canal (sc) (Figs. 14A and 14B). In *P. echinoides* (Figs. 14D and 14E) and *P. scleranthus*, the stylar canal reaches the flower apex. In *P. echinoides*, no vascular bundles are found in the upper region of the style, above the nectariferous branches. In *P. scleranthus*, the vascular bundles reach only the median part of the style.

The vascular bundles of all floral parts are collateral and formed by 2–4 transport cells (Fig. 14A). The vascularization of the staminodes and the placenta were not observed in the cross-section of any of the studied species (Figs. 13E and 13F). However, in the longitudinal section, there is a vascular bundle reaching the base of each one of the staminodes (sd) (Fig. 13G).

## Discussion

### Morphological (re)interpretation

In the dimerous species of *Paepalanthus* studied here, the floral parts emerge opposite to each other in the same whorl, with no evidence of a third set of parts. The two sepals of dimerous flowers arise simultaneously during floral development. This is similar to the condition in lateral sepals of trimerous flowers, in which the adaxial median sepal develops late (Stützel, 1984; Stützel, 1990). Thus, we hypothesize that sepals of dimerous flowers of *Paepalanthus* in fact correspond to the lateral sepals of trimerous flowers.

**Figure 15:**
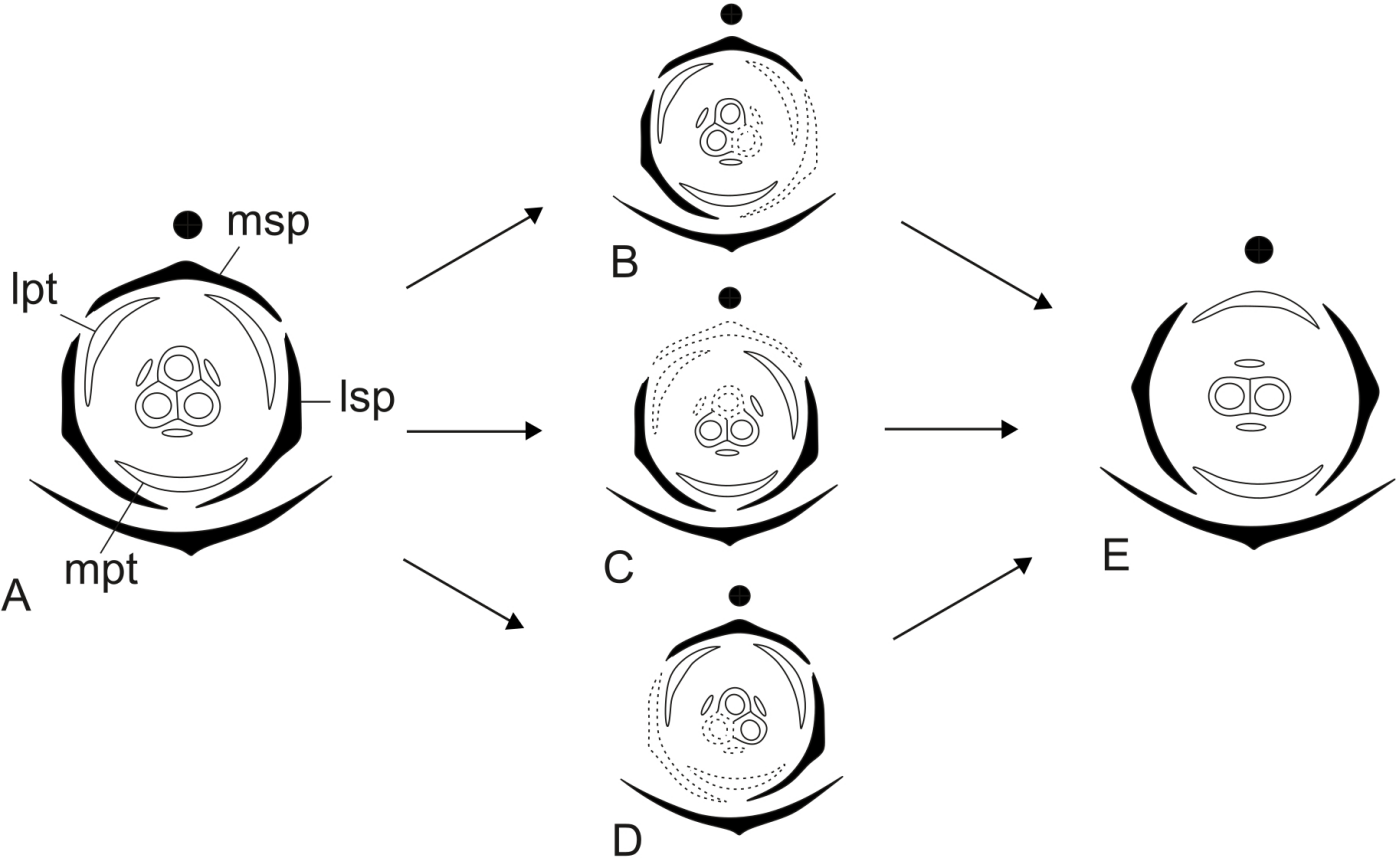
Putative steps leading to the dimerous pistillate flowers in species of *Paepalanthus*. (A) Trimerous flower. (B) Trimerous flower with a suppressed sector comprising a lateral sepal and a lateral petal. (C) Trimerous flower with a suppressed sector comprising the median sepal and a lateral petal. (D) Trimerous flower with a suppressed sector comprising a lateral sepal and the median petal. (E) Dimerous flower. Labels: lpt, lateral petal; lsp, lateral sepal; mpt, median petal; msp, median sepal.

**Figure 16:**
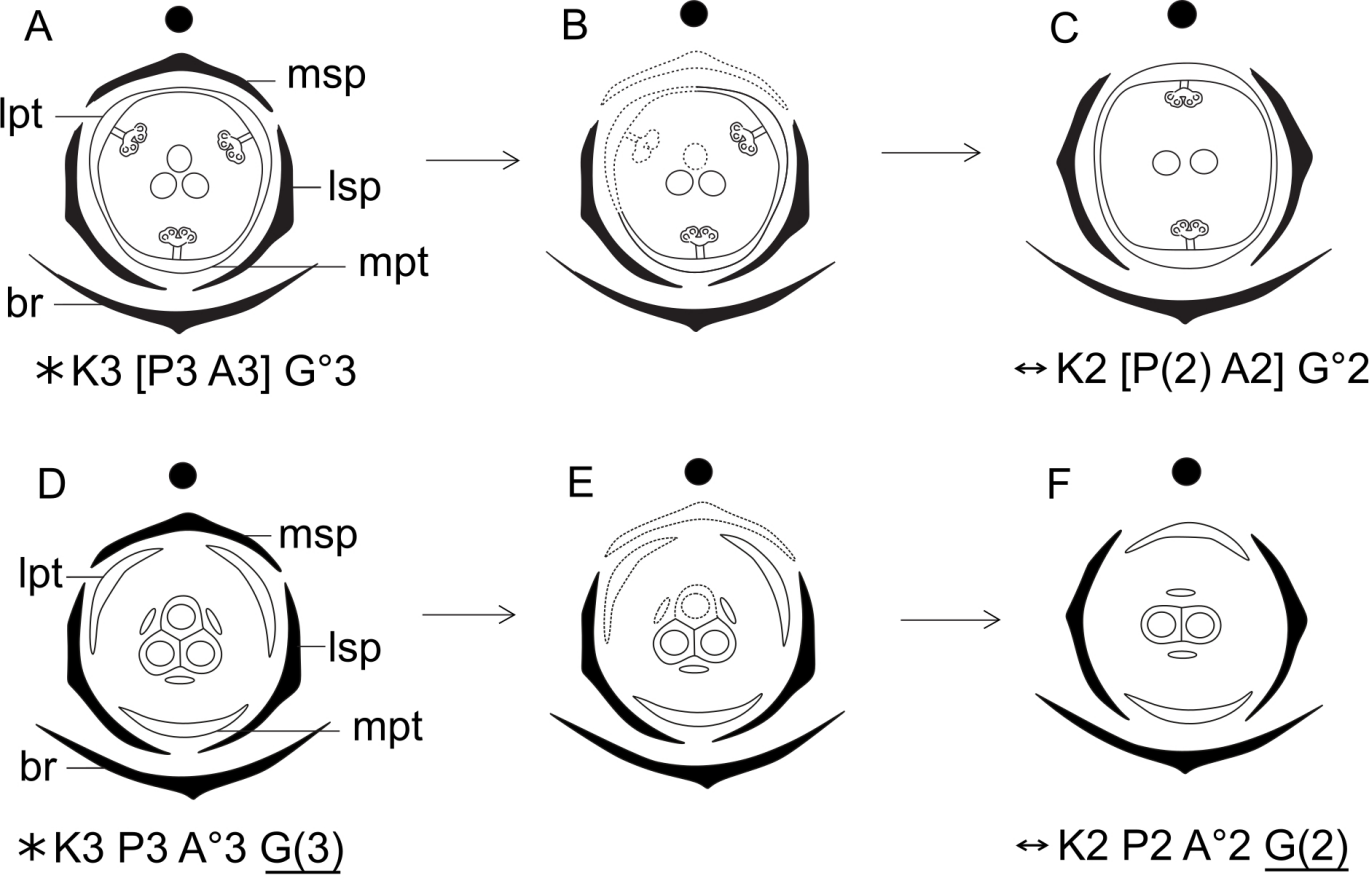
Hypothetical steps leading to the dimerous flowers in species of *Paepalanthus.* (A) Trimerous staminate flower. (B) Trimerous staminate flower with a suppressed sector comprising the median sepal, a lateral petal, a lateral stamen and the median carpel. (C) Dimerous staminate flower. (D) Trimerous pistillate flower. (E) Trimerous pistillate flower with the median sepal, a lateral petal, a lateral staminode and the median carpellode suppressed. (F) Dimerous pistillate flower. Labels: lpt, lateral petal; lsp, lateral sepal; mpt, median petal; msp, median sepal.

Dimery could have evolved through the suppression of a floral sector of trimerous flowers, given that dimerous flowers have alternating whorls, and dimery frequently occurs in families with trimerous flowers (Ronse De Craene & Smets, 1994). Meristic changes, as well as organ delays, are influenced by the size and position of the pre-existing organs (due to mechanical pressure), the size of floral meristem and the available space for the floral whorls to develop (Ronse De Craene, 2015). The perianth is also known to be important in defining and fixing the floral merism (Ronse De Craene, 2015). Regarding the minimum dislocation of floral parts after the loss of one unit of each whorl (Fig. 15), the putative interpretation for the evolution of dimery in *Paepalanthus* is of the loss of a sector comprising the median sepal, one lateral petal, one lateral stamen and the median carpel (Figs. 15 and 16). In this context, our results on floral development and position of mature floral parts of dimerous flowers corroborate Stützel’s (1990) hypothesis that the transition to dimery occurred from the suppression of the median sepal of trimerous flowers and help to explain merism evolution in *Paepalanthus*.

Particularly in some pistillate flowers of *P. scleranthus* (*P*. subg. *Thelxinoë*), sepals are asymmetrical, which may be considered as evidence that a median sepal was suppressed. However, sepal position in these flowers is probably related to the reduced space in the region where the young flowers are found—the centre of the capitulum—and therefore to the accommodation of these flowers in this region.

In previous research, Stützel (1990) and Stützel & Gansser (1995) suggested the absence of an outer whorl of stamens in early development stages of staminate and pistillate flowers of *Lachnocaulon*, *Paepalanthus* and *Syngonanthus*. Nonetheless, Rosa & Scatena (2003); Rosa & Scatena (2007) reported an outer whorl of staminodes, alternating with the fertile stamens, in staminate flowers of *Actinocephalus*, *Leiothrix*, *Paepalanthus* and *Syngonanthus*. Flower ontogeny of dimerous species of *Paepalanthus* presented in our study corroborates the results previously presented by Stützel (1990) and Stützel & Gansser (1995). In staminate flowers of *Paepalanthus*, the staminodes reported by Rosa & Scatena (2003); Rosa & Scatena (2007) in fact correspond to the apical portions of late-developing petals. We infer that similar structures were misinterpreted in other genera of Paepalanthoideae and require further investigation. Therefore, the results obtained here lead us to the conclusion that the common ancestor of the Paepalanthoideae probably had isostemonous flowers, and not diplostemonous flowers as concluded by Rosa & Scatena (2007).

The intricate development of nectariferous structures of staminate flowers, described here for the first time, exhibit early stages similar to those observed in the nectariferous branches of pistillate flowers of the same species. These structures are considered to be the result of gynoecium reduction and specialization for nectar production in staminate flowers and are commonly referred to as ‘pistillodes’ (Rosa & Scatena, 2007; Oriani & Scatena, 2012). However, during the development of the pistillate flowers we noticed that the lateral bulges of the gynoecium primordium correspond to the carpel walls and the central bulge corresponds to the ovary septum. In the staminate flower, the lateral bulges become nectariferous structures, whereas the central bulge remains undifferentiated. Therefore, we conclude that this nectariferous structures are actually homologous only to the carpels, not to the whole gynoecium, and should be referred to as ‘carpellodes’. This interpretation is corroborated by the previously observation that the pistillodes are vascularized by dorsal carpellary bundles (Rosa & Scatena, 2007). Furthermore, some morphological features of the nectariferous structures, such as shape and type of epidermal cells, differ in mature flowers of some dimerous species of *Paepalanthus*. These characteristics are important for species distinction and may also be an indicator of infrageneric delimitation in the genus.

During gynoecium formation in pistillate flowers of Eriocaulaceae, the ovary septum raises the central placenta, and the ovules become pendulous (Coan, Stützel & Scatena, 2010). In dimerous species of *Paepalanthus*, we observed that the septum does not fuse with the apical portion of the ovary in the mature flower, resulting in a proximal synascidiate zone and a short distal symplicate zone close to the stylar canal opening. This ovarian feature is common in angiosperms, and the occurrence of a distal symplicate zone, as the presence of commissural stigmatic branches, may be related to a regular distribution of pollen tubes in the locules, in case stigmas receive contrasting amounts of pollen grains (Endress, 2011).

Pistillate flowers of Paepalanthoideae have a branched style, with nectariferous branches in the carinal (dorsal) position and stigmatic branches in the commissural position of the ovary (Stützel, 1990; Rosa & Scatena, 2003). In mature flowers of Eriocaulaceae, the gynoecium branches are free and inserted at the same point on the style in most genera (Stützel, 1990). The same organization was reported for the dimerous species of *Paepalanthus* studied here, except for *P. echinoides* (*P*. sect. *Conodiscus*) and *P. scleranthus* (*P*. subg. *Thelxinoë*). The stigmatic branches in these species are short and inserted terminally on a long style. The stigmatic branches divide into two bifid stigmas, which consequently are placed close together and may be misinterpreted as four simple stigmas. However, the presence of bifid stigmas is a common condition in *Paepalanthus* (Ruhland, 1903), whereas four simple stigmas is a character state that is absent from Eriocaulaceae as a whole.

In *P. scleranthus*, pistillate flowers generally have stylar and stigmatic branches, but no nectariferous branches. In Paepalanthoideae, pistillate flowers with only stigmatic branches were verified in a few species of *Paepalanthus* and *Syngonanthus* (Ruhland, 1903; Stützel, 1987; Stützel & Gansser, 1995). The style formation in *P. scleranthus* occurs through intercalary growth, resulting in a columnar structure similar to that found in the gynoecium of *P. echinoides*. In early development of the style of *P. scleranthus*, nectariferous branch primordia are found in some pistillate flowers, but they generally do not develop, indicating this nectariferous structure was present in the species’ ancestor.

### Vascularization and homologies

Anatomical details of dimerous flowers of *Paepalanthus* showed that there is no vestige of vascularization of a third floral part in any of the floral whorls. The vascular bundles were also dislocated following the dislocation of the floral parts during the transition from trimery to dimery.

The vascularization of the staminate flower shows the presence of a petal-stamen complex. Vascular traces shared by petals and stamens are widespread among monocots and were also observed in species of *Xyris* (Xyridaceae, Poales) (Endress, 1995; Sajo, Wanderley & Menezes, 1997; Remizowa et al., 2012). In *Xyris* (which has bisexual flowers) and in staminate flowers of *Paepalanthus*, the vacularization shared by these whorls is probably related to these species’ development, as these structures emerge from common primordia. In pistillate flowers of *Paepalanthus*, staminodes share vascular traces with petals due to the homology of the staminodes and the functional stamens of staminate flowers.

The vascularization of the gynoecium of dimerous species of *Paepalanthus* is similar to that reported in previously studied species of Paepalanthoideae (Rosa & Scatena, 2003; Rosa & Scatena, 2007). However, the ventral bundles of the ovary in dimerous species are in the commissural position. Despite the position of the ventral bundles, they reach only the synascidiate portion of the ovary, and the stigmatic branches in these species lack vascularization, as is usual for trimerous species in the subfamily (Rosa & Scatena, 2003; Rosa & Scatena, 2007). In *P. echinoides* and *P. scleranthus*, the dorsal carpel bundles reach the median region of the style, whereas its upper region is not vascularized. We assume that the proximal region of the style of both species is homologous to the short style found in the other species of *Paepalanthus* studied here, which is vascularized. On the other hand, the upper region of the style is probably homologous to their stigmatic branches, which are non-vascularized. Thus, we can interpret the long style of *P. echinoides* and *P. scleranthus* as a short style on which are inserted two fused stigmatic branches.

### Evolutionary and taxonomic implications

**Figure 17:**
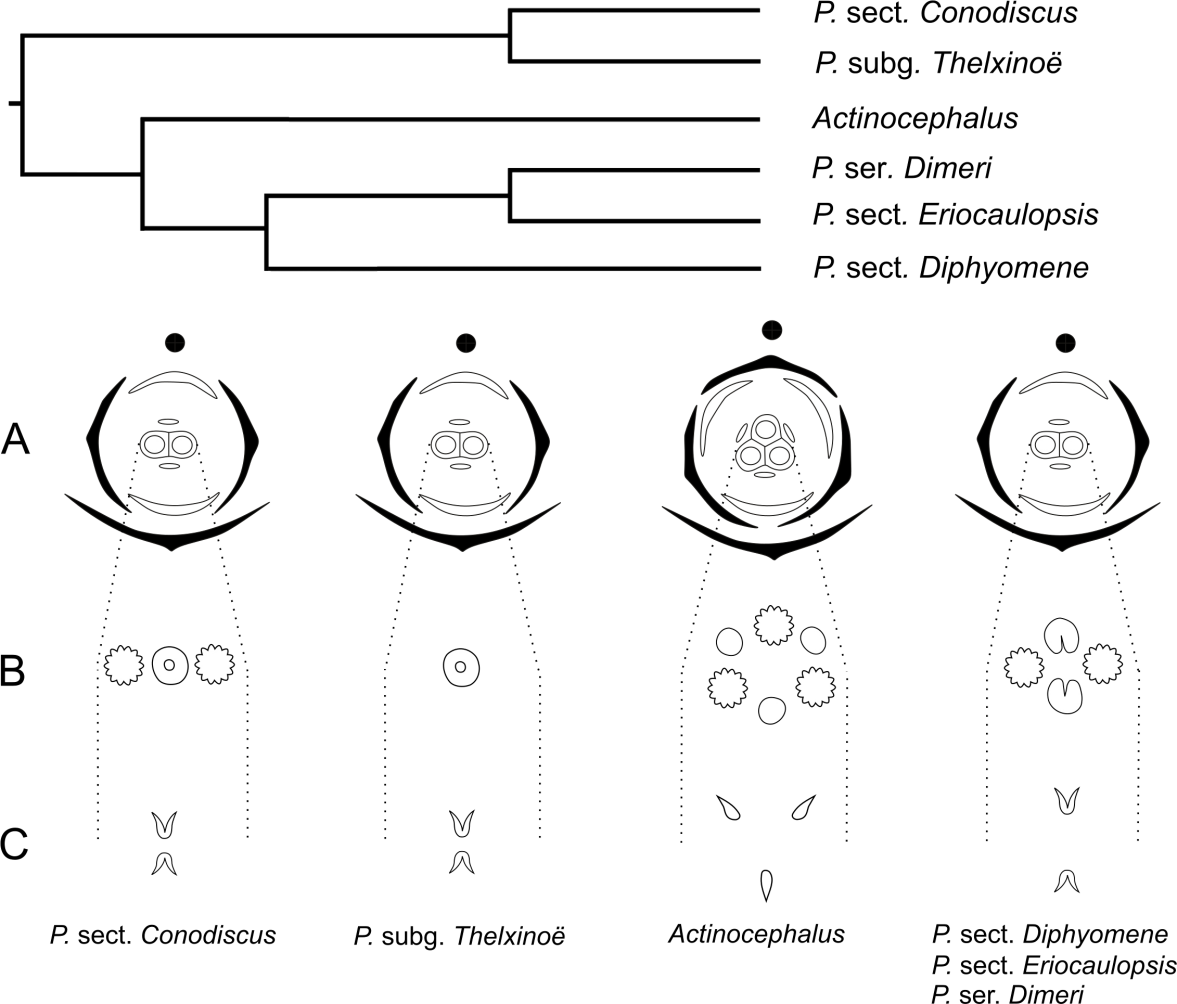
Phylogenetic relationships (adapted from Trovó et al., 2013) of the dimerous infrageneric categories of *Paepalanthus* and respective tridimensional floral diagrams of pistillate flowers compared at different levels of gynoecium (A, ovary; B, gyonoeci; C, stigma).

In recent phylogenetic studies, dimerous species of *Paepalanthus* are placed in five infrageneric categories, forming two distinct clades (Giulietti et al., 2012; Trovó et al., 2013). *Paepalanthus* subg. *Thelxinoë* appears as a sister group of *P*. sect. *Conodiscus*, and both categories together form a sister group of the clade that includes *Actinocephalus* as sister group of *P*. sect. *Diphyomene, P.* ser. *Dimeri* and dimerous species of *P*. sect. *Eriocaulopsis* (previously circumscribed in *P*. sect. *Diphyomene* (Trovó & Sano, 2010; Trovó et al., 2013) (Fig. 17). The occurrence of fused stigmatic branches in *P. scleranthus* (*P*.subg. *Thelxinoë*) and *P. echinoides* (*P*. sect. *Conodiscus*) corroborates the phylogenetic proximity of *P*.subg. *Thelxinoë* and *P*. sect. *Conodiscus* (Fig. 17). Furthermore, the rise of nectariferous branch primordia and their subsequent suppression in *P. scleranthus* indicate that nectariferous branches may have been lost after the fusion of stigmatic branches in the clade that groups together *P*. subg. *Thelxinoë* and *P*. sect. *Conodiscus* (Trovó et al., 2013).

The morphology of the papilla in the carpellodes and their exposure in male anthesis is congruent with the topology found by Trovó et al. (2013) (Fig. 17). Although species of the clade *P*. subg. *Thelxinoë* and *P*. sect. *Conodiscus* have protected carpellodes with few prominent papillae, species of the clade *P*. sect. *Diphyomene, P.* ser. *Dimeri*, and *P*. sect. *Eriocaulopsis* have exposed carpellodes with well-developed nectariferous papillae. These characteristics are probably synapomorphies of these clades and may be related to distinct pollination syndromes in these groups. The nectariferous branches are exposed above the sterile floral parts in pistillate flowers, like the carpellodes in staminate flowers, and facilitate the exposure of resources to pollinators as well as the exposure of the receptive stigmatic surface to pollen grains. *Paepalanthus scleranthus* was the only species we studied that lacks nectariferous branches in the gynoecium and may have an abiotic pollination syndrome, distinct from the previously studied species of Eriocaulaceae (Ramos, Borba & Funch, 2005; Oriani, Sano & Scatena, 2009).

For a more general panorama of floral characters in a phylogenetic context, we must expand our knowledge about the flower ontogeny in *Actinocephalus* and its relationship to floral characters of species of *Paepalanthus* sect. *Diphyomene, P.* ser. *Dimeri* and dimerous species of *P*. sect. *Eriocaulopsis.* Despite the suggestion that dimery has evolved more than once in *Paepalanthus,* this study reveals a possible alternative interpretation—that dimerous flowers have appeared only once, followed by a reversal in *Actinocephalus* (Fig. 17). It also seems clear that, although morphologically distinct, the exclusion of dimerous species from *P*. sect. *Diphyomene* (Trovó & Sano, 2010) may be reconsidered after new evidence.

## Final Remarks

The early development stages of flowers in the dimerous species of *Paepalanthus* studied here are similar to reports by Stützel (1990) and Stützel & Gansser (1995) for other Eriocaulaceae species. However, some development aspects and vascularization details of dimerous flowers of *Paepalanthus* are new and important additions to the knowledge of the whole family. The results presented here contribute to the understanding of floral merism evolution in *Paepalanthus*, a genus of Eriocaulaceae whose morphology is unusually complex. In addition to corroborating the hypothesis proposed by Stützel (1990) for dimery evolution in Eriocaulaceae, the sepal development, the position of floral parts, and the vascularization of dimerous species of *Paepalanthus* also helped to determine the position of the suppressed floral parts in each floral whorl. Furthermore, the correct interpretation of the incipient petal in staminate flowers of *Paepalanthus*, and the consequent absence of staminodes in these flowers, reinforce the supposition that the ancestor of Paepalanthoideae had isostemonous flowers, rather than diplostemonous flowers as was inferred by previous anatomical studies in the family (Rosa & Scatena, 2003; Rosa & Scatena, 2007). Finally, the data obtained here show the importance of comparative ontogenetic studies for developing a better understanding of floral structures and their evolution in Eriocaulaceae.

## Additional Information and Declarations

### Field Study Permissions

The following information was supplied relating to field study approvals (i.e., approving body and any reference numbers): The examined material is listed in Table 1 based on the following field permits: SisBio collecting permit no. 47742-1 (February 2015–March 2016) to AL Silva; SisBio permanent permit no. 34782-1 (from May 2012 to present) and collecting permit no. 42939-1 (May 2014–June 2015) to M Trovó; and SisBio permanent permit no. 14929-3 (December 2011 to present) and collecting permits no. 37568-1 and 37568-2 (January 2013–February 2015) to AI Coan.

### Data Availability

The following information was supplied regarding data availability: The raw data is included in Table 1.
